# Longitudinal qPCR Study of the Dynamics of *L. crispatus*, *L. iners*, *A. vaginae*, (Sialidase Positive) *G. vaginalis*, and *P. bivia* in the Vagina

**DOI:** 10.1371/journal.pone.0045281

**Published:** 2012-09-21

**Authors:** Guido Lopes dos Santos Santiago, Inge Tency, Hans Verstraelen, Rita Verhelst, Marijke Trog, Marleen Temmerman, Leen Vancoillie, Ellen Decat, Piet Cools, Mario Vaneechoutte

**Affiliations:** 1 Laboratory for Bacteriology Research, Department of Clinical Chemistry, Microbiology, and Immunology, Faculty of Medicine and Health Sciences, Ghent University, Ghent, Belgium; 2 Department of Obstetrics and Gynaecology, Faculty of Medicine and Health Sciences, Ghent University, Ghent, Belgium; 3 AIDS Reference Laboratory, Department of Clinical Chemistry, Microbiology, and Immunology, Ghent University/Ghent University Hospital, Ghent, Belgium; 4 Faculty of Health Care, University College Ghent, Ghent, Belgium; University of Iowa Carver College of Medicine, United States of America

## Abstract

**Background:**

To obtain more detailed understanding of the causes of disturbance of the vaginal microflora (VMF), a longitudinal study was carried out for 17 women during two menstrual cycles.

**Methods:**

Vaginal swabs were obtained daily from 17 non-pregnant, menarchal volunteers. For each woman, Gram stains were scored, the quantitative changes of 5 key vaginal species, *i.e. Atopobium vaginae*, *Lactobacillus crispatus*, *L. iners*, (sialidase positive) *Gardnerella vaginalis* and *Prevotella bivia* were quantified with qPCR and hydrogen-peroxide production was assessed on TMB+ agar.

**Results:**

Women could be divided in 9 subjects with predominantly normal VMF (grades Ia, Ib and Iab, group N) and 8 with predominantly disturbed VMF (grades I-like, II, III and IV, group D).

VMF was variable between women, but overall stable for most of the women. Menses were the strongest disturbing factor of the VMF.

*L. crispatus* was present at log7–9 cells/ml in grade Ia, Iab and II VMF, but concentrations declined 100-fold during menses. *L. crispatus* below log7 cells/ml corresponded with poor H_2_O_2_-production. *L. iner*s was present at log 10 cells/ml in grade Ib, II and III VMF. Sialidase negative *G. vaginalis* strains (average log5 cells/ml) were detected in grade I, I-like and IV VMF. In grade II VMF, predominantly a mixture of both sialidase negative and positive *G. vaginalis* strains (average log9 cells/ml) were present, and predominantly sialidase positive strains in grade III VMF. The presence of *A. vaginae* (average log9 cells/ml) coincided with grade II and III VMF. *P. bivia* (log4–8 cells/ml) was mostly present in grade III vaginal microflora.

*L. iners*, *G. vaginalis*, *A. vaginae* and *P. bivia* all increased around menses for group N women, and as such *L. iners* was considered a member of disturbed VMF.

**Conclusions:**

This qPCR-based study confirms largely the results of previous culture-based, microscopy-based and pyrosequencing-based studies.

## Introduction

The vaginal microflora (VMF) has been studied extensively and has been shown to be easily disturbed by exogenous and endogenous factors [1]–[3]. Still, the etiology of bacterial vaginosis, a polymicrobial condition whereby the lactobacilli-dominated VMF is replaced largely by anaerobes, remains to be elucidated [4] and the general dynamics of the VMF remain to be understood.

Several longitudinal studies of the VMF throughout the menstrual cycle (MC), as a tool for understanding the dynamics of the VMF, have been undertaken. Most of these studies relied on analysis of Gram stained smears using the Nugent score [5]–[8], and some studies combined this with culture of vaginal swabs [9]–[11], and very recently Gajer *et al.* [12] combined pyrosequencing with Gram stained smears, but to our knowledge only one group [13] previously combined Gram stained smears with qPCR for analysis.

The present study builds upon previous work of our group, where we reported the results of Gram stain and culture for 17 menarchal women, who self-swabbed the vagina daily during two MCs [10]. Here, we add concentrations obtained by quantative PCR for five vaginal key species, *i.e. Atopobium vaginae*, *Lactobacillus crispatus*, *L. iners*, *Gardnerella vaginalis* and *Prevotella bivia*, with *L. crispatus* being the predominant *Lactobacillus* in normal VMF [14]–[16], *L. iners* being the single most predominant *Lactobacillus* species in bacterial vaginosis associated VMF in all of our previous studies [10], [14], [17], but also an important species in normal VMF, according to other studies [18]–[20]. *A. vaginae* and *G. vaginalis* being important markers for BV [17], [21]–[24] were also assessed. qPCR was also performed for the *G. vaginalis* sialidase gene, since the presence of sialidase is considered as an indicator for BV and preterm birth (PTB) [25]–[28]. Moreover, we found that there is a clear genotypic distinction between sialidase producing and sialidase negative *G. vaginalis* strains [29]. Finally, qPCR for the *P. bivia* mucin-desulfating sulfatase gene (mdsC) was developed, because the activities of sialidase and sulfatase, both produced by *P. bivia* [30], have been suggested to be rate-limiting in the breakdown of the mucin layer and thus to be important factors in the pathogenesis of BV [30]–[33]. Moreover, some studies have correlated the presence of *P. bivia* in vaginal fluid with an important increase in PTB [34], [35] and *P. bivia* has also been shown to enhance the growth of *G. vaginalis* through ammonia production [36].

In addition, we also assessed the presence of vaginal microorganisms producing hydrogen peroxide by culture. Some *Lactobacillus* strains have been shown to produce H_2_O_2_, which has been generally accepted as an important defense mechanism against vaginal colonisation by pathogens and a lack of vaginal H_2_O_2_-producing lactobacilli has been associated with the acquisition of BV [37]–[39].

Briefly, the general objectives of this study were to assess the presence and to quantify *L. crispatus* and *L. iners* during the menstrual cycle, also in relation to the BV-associated species, to assess the presence and concentrations of sialidase positive strains of *G. vaginalis* in normal and disturbed VMF, to assess the influence of the menstrual cycle and sexual intercourse on the presence and concentrations of the 5 species, and finally to add complementary information to the culture study previously performed [10] on this study population.

## Materials and Methods

### Ethics Statement

This study was approved by the research ethics committee (EC UZG 2008/439) of the Ghent University Hospital (GUH), Belgium.

### Subjects

Twenty-five female volunteers, aged between 18 and 35 years, were recruited after oral and written informed consent. The inclusion criteria were a regular MC and no use of contraception, except condoms. The exclusion criteria were pregnancy, complaints about malodorous vaginal discharge, recent history of vulval irritation, chronic use of medication, the use of antibiotics, antimycotics and antiprotozoals during the past two months, a history of vaginal surgery or hysterectomy, pelvic inflammatory disease, recurrent vaginal infections, an active vulval or vaginal dermatological aberration, vaginal douching within the last week before the study and symptomatic candidiasis or a positive *Chlamydia trachomatis* PCR result. After screening, 22 women were included, of which 17 completed the study.

### Study design

The participants were asked to take vaginal swabs (ESwab, Copan, Brescia, Italy) each day of the study. The swabs were stored at 4°C and once a week all swabs were transported to the GUH.

A Gram stain was made from a smear of each vaginal swab. Only the last swab of each week, taken on the day of transport to the GUH, was cultured anaerobically on Schaedler agar, Colombia agar and TMB^plus^ agar [10]. From a selection of swabs (see below), 200 µl (per swab) was used for DNA-extraction with the EasyMag platform (bioMérieux, Marcy l'Etoile, France).

**Figure 1:**
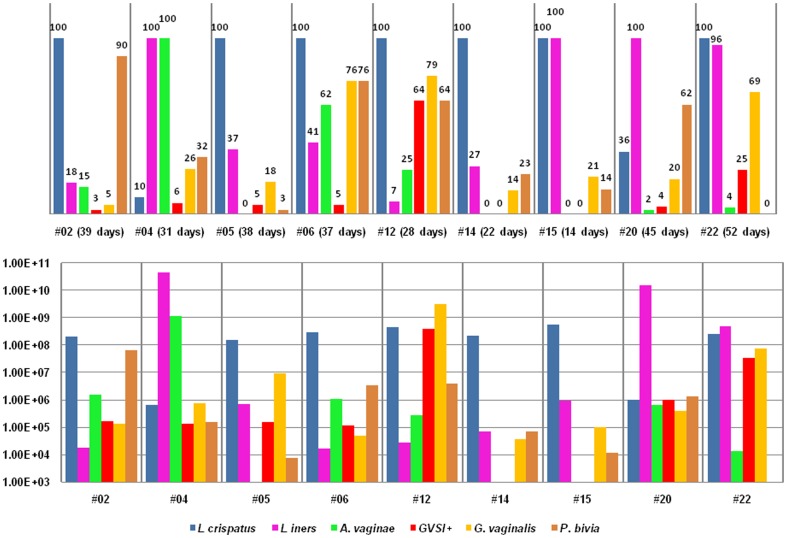
The presence and average concentrations of the 5 species during the study in group N. Legend: The chart above represents the total presence (%) of the 5 species during the days that were analysed. In addition, the actual days present versus the total days analysed are also given. The chart below represents the average concentrations (cells/ml) of the 5 species during the days that were analysed.

**Figure 2:**
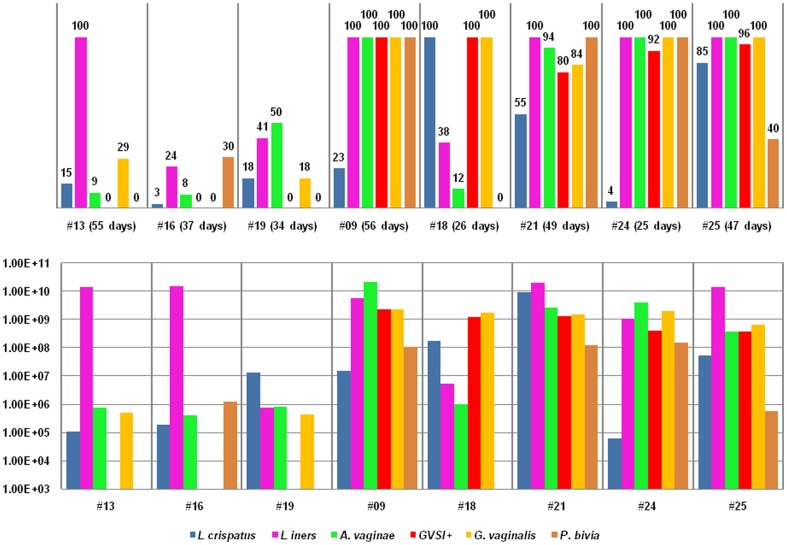
The presence and average concentrations of the 5 species during the study in group D. Legend: The chart above represents the total presence (%) of the 5 species during the days that were analysed. In addition, the actual days present versus the total days analysed are also given. The chart below represents the average concentrations (cells/ml) of the 5 species during the days that were analysed.

To limit the workload and cost of the study, DNA-extraction and qPCR were carried out only on a selection of the samples, according to the following criteria: each day of the menses, two days before and two days after the menses, two days before and after a change of the grade Ia VMF (see below), as established based on Gram stain of the vaginal swab, every other day during the change in VMF, on the day of sexual intercourse, each day that the VMF remained changed after the sexual intercourse, and until two days after the VMF restored its previous status. The number of days that were assessed per volunteer varied from 14 days (#15) to 56 days (#9) (Figures 1 and 2).

### Grading of Gram-stained vaginal smears

The Gram stained vaginal smears were scored by four independent assessors according to previously described modified Ison and Hay criteria [10], [14]. For reference, grades Ia, Iab, Ib and I-like correspond with Nugent score 0–3, grade II and IV with Nugent score 4–6 and grade III with Nugent score 7–10. However, in the modified Ison and Hay criteria, grade I-like is not considered as normal VMF [14] and the position of grade Ib as normal VMF is questionable [10].

### DNA-extraction from swab medium

For DNA-extraction, the ESwab medium obtained from each vaginal swab was pretreated. A total volume of 200 µl of the E-swab medium was transferred to a 2 ml tube, to which 200 µl of buffer (20 mM Tris-HCl, pH 8.0, 0.5% SDS) was added. Subsequently, 2 µl of mutanolysin (25 U/µl) was added and the mixture was incubated for 15 min at 37°C. Next, 10 µl of a 25 mg/ml proteinase K solution was added and the mixture was incubated for 15 min at 55°C. Finally, NucliSENS EasyMAG lysis buffer was added to a final volume of 2 ml, and incubated for 10 min at room temperature. Pretreated swab medium was stored at −80°C until DNA extraction. DNA extraction was performed on the NucliSENS EasyMAG (BioMérieux) platform, according to the manufacturer's instructions.

### qPCR

qPCR assays for *Lactobacillus crispatus*, *L. iners*, *Atopobium vaginae*, *Gardnerella vaginalis*, and *G. vaginalis* sialidase gene were carried out as described previously [17], [29].

The sequence of the mucin-desulfating sulfatase gene (*mdsC*, NZ_ADFO01000048) from the partially sequenced *P. bivia* strain JCVIHMP010 (reference genome for the Human Microbiome Project, J. Craig Venter Institute, San Diego, CA) served as the template for designing the primer set for amplification of the *mdsC* gene from *P. bivia*. Primer Blast (NCBI) was used to design the following primer set: PBsulF (5′ ACGTTTGGGCAAAGCTCCTTGTCT) and PBsulR (5′GCGTGTACGCCAGTTGCAAGA). Annealing of primers on secondary structures was analysed using mFOLD (http://mfold.rna.albany.edu/?q=mfold). Quantification of the *P. bivia* mucin-desulfating sulfatase gene was carried out after amplification with the LC480 SYBR Green® I master kit (Roche, Basel, Switzerland) on the Light Cycler 480 real-time PCR system (Roche, Basel, Switzerland).

Jellyfish DNA was used as an internal inhibition control, whereby a difference of 2 Cq or more was used as a cut-off to classify a sample as having significant qPCR inhibition [40].

### Hydrogen-peroxide-production

The hydrogen-peroxide-production was assessed by direct inoculation of 75 µl of ESwab medium onto TMB^plus^ agar plates [41]. After a two day incubation period at 37°C in an anaerobic chamber (BugBox, LedTechno, Heusden-Zolder, Belgium), the plates were exposed to oxygen for 1.5 hours and photographed. Each plate was given a score according to the intensity of the blue color: no blue color = score 0 (no H_2_O_2_ production), score 1 (weak H_2_O_2_-production), score 2 (strong H_2_O_2_-production) and score 3 (very blue, very strong H_2_O_2_-production) [42].

### Statistical analysis

The statistical analysis of the results was performed with the Wilcoxon signed-rank test and the Mann–Whitney U test.

## Results

Based on Gram stained vaginal smears, the 17 volunteers could be allocated into two groups, whereby group N(ormal) (n = 9) comprised the women with a predominantly stable normal VMF (grades Ia, Iab and Ib) and group D(isturbed) (n = 8) comprised the women with predominantly disturbed VMF (grades I-like, II, III and IV, *i.e.* non-grade I). Within group N, two women could be set further apart from the other 7 subjects, because they were the only two in group N with primarily grade Ib VMF, with *L. crispatus* only sporadically present.

Jellyfish DNA qPCR was carried out for DNA extracts of vaginal swabs taken during 7 menses periods of 7 subjects. DNA-extracts (including a 100-fold dilution) taken from 4 days before the menses, during the menses and 4 days after the menses were tested for qPCR inhibition. No inhibition could be observed.

File S1 depicts an overview of the qPCR results, representing the bacterial concentration of the five species tested and the concentration of the *G. vaginalis* sialidase gene, during the study period (two menstrual cycles) for each of the 17 women, with indication of the following parameters for each sampling moment: the VMF grade (according to Gram stain), the presence of gram positive cocci (GPC) and of yeast cells on Gram stain, the H_2_O_2_ production score, occurrence of menses and of sexual intercourse, usage of antibiotics and the hygienic practices.

### Bacterial concentrations during the menstruation cycle

#### *Lactobacillus crispatus*

In group N, 7 of the 9 women had high concentrations of *L. crispatus* (average log7-log9 cells/ml). For two women of group N with primarily grade Ib VMF (#4, #20), *L. crispatus* was only sporadically present, but *L. iners* was present in high concentrations.

For 6 of the 7 women with predominantly *L. crispatus*, the concentrations of this species declined significantly, *i.e.* 100-fold on average, during the menses .

Within group D, different types of disturbances of the VMF and different prevalences of *L. crispatus* were observed. The two women (#13 and #19) with a VMF shifting between grades Ib, II, I-like and IV VMF, were sporadically colonized by *L. crispatus* with an average bacterial concentration of log6 cells/ml. The two subjects (#18 and #25), with predominantly grade II VMF were characterized by high concentrations of *L. crispatus* (log5-log9 cells/ml), and – as for group N subjects with high a *L. crispatus* concentration, there was a 100-fold decrease during the menses. The two subjects with grade III, *i.e.* BV-associated VMF (#9 and #24), had no or very low concentrations of *L. crispatus*.

#### *Lactobacillus iners*

For five out of 9 group N women, *L. iners* was found only around and during the menses, and only in low concentrations (log4-log6 cells/ml). In 2 women (#15 and # 22), *L. iners* and *L. crispatus* were present as a mixture during all days that were assessed. The women (#4 and # 20) with primarily grade Ib VMF carried high concentrations of *L. iners* (log10 cells/ml on average).

In Group D, the women (#9, #24) with predominantly grade III VMF and the women that shifted regularly between the grades Ib, II, III and IV (#13, #21, #25 ) had high concentrations of *L. iners* (log10 cells/ml), throughout the MCs. For 2 women (#18, #19) with constantly grade II and grade I-like/IV, respectively, *L. iners was* sporadically present, particularly just before, during and after the menses with concentrations varying between log5 and log6 cells/ml.

#### *Gardnerella vaginalis*

In general, there was an increase in *G. vaginalis* concentrations when the VMF changed from normal to disturbed, and vice versa a decrease, when the disturbed VMF shifted to normal VMF (*e.g.* before, during and after the menses). More specifically, in Group N, all women carried *G. vaginalis* at a certain point in time (concentrations ≤log5 cells/ml ). *G. vaginalis* sialidase positive strains were virtually absent in Group N.

*G. vaginalis* was present in 7 of the 8 women of group D and was only absent in one of the two subjects that used antibiotics. The two women (#13, #19) with a VMF shifting between grades Ib/II/IV and grade I-like/IV VMF respectively, had *G. vaginalis* (sialidase negative strains only) concentrations lower than log6 cells/ml. Grade II and III VMF were characterized by *G. vaginalis* sialidase positive strains, mostly as a mixture together (minimum log8 cells/ml) with sialidase negative strains.

#### *Atopobium vaginae*

*A. vaginae* was present in 6 of 9 women in Group N, but in 4 women only sporadically (log5 cells/ml on average) and mostly around the menses and/or when the VMF was disturbed (grade II). *A. vaginae* colonized two subjects during the entire study (average log9 cells/ml and log6 cells/ml respectively), with a 10 to 100-fold increase in bacterial concentrations during the menses.

In Group D, half of the women, *i.e.* the women shifting between grades Ib/II/III or with continuous grade III VMF, had high concentrations of *A. vaginae* (log9 cells/ml average). The two subjects (#9, #24) with a continuous grade III VMF had a significantly higher (p<0.001) concentration of *A. vaginae* in comparison to *G. vaginalis*. In the other four group D women, for whom no or almost no grade III VMF episodes could be observed, *A. vaginae* was only sporadically present, with low average concentrations, varying between log4 and log5 cells/ml.

#### *Prevotella bivia*

In Group N, 8 of 9 women were colonized by *P. bivia* around the menses and when the VMF was disturbed (grade I-like and II). Outside the menses, the concentrations of *P. bivia*, when present, varied between log4 and log6 cells/ml. During the menses, there was a 1–2 log increase, but it was only significant for one subject (p = 0.036). When the VMF remained disturbed after the menses (grade II VMF) or when the VMF shifted to grade II VMF, the concentrations increased to log9 cells/ml.

In Group D, 5 of 8 women, *i.e.* the women with an overall grade Ib/II/III or continuous grade III VMF, were colonized by *P. bivia* (average 7 log7 cells/ml). For 4 of these women, the concentration increased by 1–2 logs during menses, but only significantly in one subject (p = 0.038). The woman (#18), with continuous grade II VMF during the study, was devoid of *P. bivia*, and the same was true for the subjects with grade Ib/II/III VMF, when they had episodes of grade II VMF.

### Comparison of the presence of the 5 species in relation to each other

Within group N, *L. crispatus* was present on all tested days for all women (Figure 1), except for the two subjects with primarily grade Ib (#4 and #20). In group N, the daily presence of continuous high concentrations of *L. crispatus* (average log8 cells/ml) appeared to be protective against the prolonged presence of *A. vaginae*, *P. bivia* and *G. vaginalis* (sialidase positive and negative) (Figure 1). The two women with the most stable grade I VMF (almost no shifts between grades), *i.e.* subjects #14 and #15 (woman with no menses), had no *A. vaginae* and no sialidase positive *G. vaginalis* during the study and *L. crispatus* (#14) or *L. crispatus* and *L. iners* (#15) were the only species of the 5 tested that were constantly present.

In group D (Figure 2), *L. iners* (average log9 cells/ml), *A. vaginae* (average log9 cells/ml), *G. vaginalis* (average log 9 cells/ml), sialidase positive *G. vaginalis* (average log8 cells/ml) and *P. bivia* (average log8 cells/ml) were present on all tested days of the two women (#9 and #24) with a continous grade III BV-associated VMF. In addition to these 4 species, the subjects (#21 and #25) shifting between grades Ib, II, III and IV VMF also had high concentrations (minimum log7 cells/ml) of *L. crispatus*, although *L. crispatus* was not present on all days. For subject #18, with grade II VMF throughout the study, all tested days were characterized by the presence of *L. crispatus* (average log8 cells/ml) and sialidase negative and sialidase positive *G. vaginalis* strains (average log9 cells/ml).

### Hydrogen-peroxide-production

For 8 out of 9 women of group N, strong (score 2) to very strong (score 3) H_2_O_2_-production was observed. *L. crispatus* concentrations higher than log7 cells/ml characterized the VMF of all of these women . For the subjects, where a significant decrease in *L. crispatus* concentrations during the menses was observed, the H_2_O_2_-production was only negatively affected when the concentrations dropped below log7 cells/ml, irrespective of the VMF grade. Exceptions were observed for subject #22 on two occasions, whereby the *L. crispatus* concentration remained high, but the H_2_O_2_-production dropped and for subject #20, with low concentrations of *L. crispatus* during the complete study period (average log6 cells/ml), but strong H_2_O_2_-production. However, the latter subject was colonized by *L. jensenii* during the whole study, as determined by culture.

In group D, 5 out of 8 women had none to very weak (score 0–1) H_2_O_2_-production. These women were characterized by *L. crispatus* concentrations below log7 cells/ml. The three subjects with *L. crispatus* concentrations higher than log7 cells/ml had a strong H_2_O_2_-production.

In general, when the concentrations of *L. crispatus* were below log7 and/or *L. jensenii* was absent, no or only weak H_2_O_2_-production was observed.

The culture results confirm the importance of *L. crispatus* and *L. jensenii*. From a total of 178 culture moments, 108 moments presented with blue colonies on TMB+ agar, 73 of these 108 culture moments had a H_2_O_2_ score >1. *L. crispatus* and *L. jensenii* were present on respectively 36 and 40 of these 73 culture moments. In comparison, Streptococci where only present on 14 of these 78 culture moments. No *L. crispatus* was cultured from the 35 culture moments with a H_2_O_2_-score<1.

File S2 contains detailed information about all species that colored blue on TMB+ agar, together with the culture moment and the corresponding H_2_O_2_ score and VMF grade.

### The influence of individual behavior on the presence of the 5 species

In general, there was no clear influence of personal hygiene (bathing, shower, intimate hygiene, tampon use) on the presence of the 5 species.

In group N, 7 incidences of sexual interaction (SI), of which 5 assessable, were documented for two subjects. For one subject, *A. vaginae* appeared (at <log6 cells/ml) after SI with condom and the concentration of *P. bivia* increased (with 4 logs). For the other subject, after SI with condom, *G. vaginalis* appeared with concentrations between log5 and log6 cell/ml.

In group D, 40 incidences of SI were documented for 4 subjects. However, because the VMF was already non-grade I or because SI occurred during the menses, only 7 instances of SI from one subject could be assessed. In this case, SI without condom resulted in the appearance of *A. vaginae* and *G. vaginalis* (sialidase negative) with concentrations between log5 and log6 cell/ml.

### The influence antibiotic therapy on the presence of the 5 species

Subject #16 started the study with only *L. iners*, at log10–log11 cells/ml, and when the 5-day antibiotics cure was initiated, *L. iners* disappeared quickly. *A. vaginae* was present on one day during the antibiotics cure and it reappeared after the cure on two more occasions. *P. bivia* was not present before the antibiotic treatment, but it was the first species to appear 22 days after the treatment. *L. iners* reappeared only 30 days later at the end of the study.

Subject #21 followed a 9-day antibiotics cure, whereby all 5 species remained present during the first 3 days. Thereafter, *L. crispatus* disappeared completely and only reappeared 3 weeks later. The concentrations of the other 4 species decreased more than 2 log, but these species recovered quickly.

## Discussion

### Overall findings

In this study, *L. crispatus* was present in high concentrations when the VMF on Gram stain was grade Ia, Iab or II, and the concentrations clearly declined during the menses of the nine women, who were predominantly colonized by *L. crispatus* (7 from group N and 2 from group D). These results confirm the findings from previous studies [10], [13], [16]. The BV-associated bacteria, *G. vaginalis*, *P. bivia* and *A. vaginae* appeared to be constituents of the normal vaginal community in our study population, but remained suppressed, increasing in numbers predominantly around menses or because of other disturbances, in association with a decline of *L. crispatus*. Indeed, around and during the menses, when the *L. crispatus* concentrations decreased, *G. vaginalis* (9 of 9 women), *A. vaginae* (6/9), *P. bivia* (8/9) and *L. iners* (6/9) appeared or their levels increased several logs.

In general, the presence of *L. crispatus* on all analyzed days, in combination with its numerical dominance, appeared to be protective against disturbance of grade I VMF and the two women with the most stable grade I VMF appeared to have a lower species diversity than the other subjects (as determined by culture [10] and qPCR (this study)).

In the present study, concentrations of *L. crispatus* below log7 cells/ml corresponded in general with poor H_2_O_2_-production. One exception was the subject that was colonized by *L. jensenii* (a strong H_2_O_2_-producer [43]) throughout the follow up period, as determined in our previous culture-based study [10]. It has been hypothesized that H_2_O_2_ production by *L. crispatus* and *L. jensenii* strongly contributes to colonisation resistance [44], [45]. However, recent studies by O' Hanlon *et al.* [46], [47] have shown that cervicovaginal fluid and semen have a significant H_2_O_2_-blocking activity and that physiological concentrations of H_2_O_2_ below 100 µM did not kill any of the tested BV-associated bacteria, *e.g. A. vaginae*, *G. vaginalis*, *P. bivia* [46], [47]. An observation supporting the findings by O' Hanlon *et al.* [46], [47], is the fact that in this study, in the two subjects with predominant grade II VMF and with *L. crispatus* concentrations above log7 cells/ml, the concomitant high H_2_O_2_ production (score 2–3) did not seem to be protective against the presence of high concentrations of BV-associated bacteria.

*L. iners*, present in all 17 women, had an almost inverse pattern compared to *L. crispatus*: in women with normal VMF, it was largely absent, or barely (only around the menses) present. Interestingly, grade Ib, thus far considered as normal VMF, was characterized by high concentrations of *L. iners* (average log10 cells/ml). In general, *L. iners* appeared to be an important constituent of grade Ib, II and III VMF, confirming earlier culture results [10] and qPCR results [17] of our group. This is not in contradiction with other studies, claiming that *L. iners* is a predominant constituent of the VMF [2], [12], [16], [48], with the remark that all of our studies [10], [14], [15], [17], [21] indicate that *L. iners* is associated with disturbed VMF, whereas others consider this species as part of the normal VMF. Compared to *L. crispatus* or *L. jensenii*, *L. iners* produces less H_2_O_2_ [49], [50] and provides relatively lower resistance to colonization to pathogens [15], [51]. In addition, Hummelen *et al.* [19] found that, when *L. crispatus* is not present in the vaginal econiche, high concentrations of *L. iners* are required to ensure a low pH. Growth assays showed that *L. iners*' preferred carbon sources are mucins, and glycosidase activity has been predicted [20]. These metabolic properties, *i.e.* haemolytic and mucinolytic activity, are indicative for a more pathogenic role of *L. iners*, enabling it to thrive in disturbed vaginal conditions, *i.e.* at high pH and in a VMF dominated by anaerobes, and confirming our repeated findings of an association of this *Lactobacillus* species with disturbed VMF. Whether or not *L.iners* can play a role in initial restoration of the VMF remains to be elucidated.

*G. vaginalis* was detected in all but one woman, but concentrations were very variable. *G. vaginalis* has been found to be present in normal VMF in several studies [10], [13], [16], [17], [19], especially around the menses. However, Roy *et al.* [52] found that symptoms that involve *G. vaginalis* were associated with >10^7^ cfu of this species per gram of vaginal fluid [52], suggesting that the presence of *G. vaginalis* below this threshold is not problematic.

In general, we found that there is a tendency for the presence of sialidase producing *G. vaginalis* strains in disturbed VMF conditions (*i.e.* grade II and III, but not grades I-like and IV), although the association is not absolute. Sialidase production has been linked to biofilm production, *e.g.* for *Streptococcus pneumoniae* and *Pseudomonas aeruginosa* (discussed in Lopes *et al.* [29]), and it has been shown that there are aggregating, biofilm producing *G. vaginalis* strains, more associated with BV, and non-aggregative, commensal *G. vaginalis* strains [53]. Whether there is a link between sialidase positive *G. vaginalis* and biofilm formation, or between sialidase producing *G. vaginalis* and (recurrent) BV or preterm birth remains to be studied.

*P. bivia* was present in 13 of the 17 women of which unexpectedly 8 out of 9 group N women, although with low concentrations (log4–log6 cells/ml), and predominantly situated around the menses or when the VMF shifted to grade I-like or grade II. In Group D, *P. bivia* was related to grade III BV-associated VMF, with concentrations of 2 to 3 logs higher in comparison to Group N. The presence of *P. bivia* was also higher around the menses.

Overall, the genus *Prevotella* has been found to be present in normal and intermediate VMF, but predominantly and in high numbers in BV [2], [16], [19].

*A. vaginae* was present in 14 of the 17 women, *i.e.* also in 6 of the 9 women from Group N, but in the latter group only occasionally, and predominantly around menses and at concentrations below log6 cells/ml. In our previous qPCR based study [17] and in other studies [16], [22], [54], *A. vaginae* was also occasionally detected in normal VMF, at low numbers, and also more prominently during BV episodes. In Group D, the presence of *A. vaginae* coincided primarily with grade III VMF. This and previous studies indicate that *A. vaginae* might be a more reliable marker for BV than *G. vaginalis* [17], [21], also because it is less frequently present than *G. vaginalis* in high concentrations in non grade III VMF, but it may be on the safe side to test for both *A. vaginae* and *G. vaginalis* to diagnose BV, as was suggested by Menard *et al.* [22].

### Comparison of qPCR and culture results

We compared our qPCR data (this study) with those obtained by culture for the same group of subjects [10]. When qPCR and culture results were combined, 3 to 4 *Lactobacillus* spp. were detected per woman in group N and 2 to 4 *Lactobacillus* spp. were detected per woman in group D.

qPCR detected *L. iners* in all 9 women of group N, but this species was cultured only in 4 women, of which the 2 subjects with grade Ib VMF. *L. crispatus* was detected in all group N women with qPCR and in all, but the two with grade Ib with culture; *A. vaginae* was present in 6 women according to qPCR but in none according to culture; *G. vaginalis* was detected in all 9 women with qPCR, but in only 3 women with culture, and *P. bivia* was detected in 8 women with qPCR, but in none with culture.

In group D, *L. iners* was detected in all 8 women with qPCR and in 5 with culture; *L. crispatus* was detected in all women with qPCR, but in only 2 women ( grade II and shifting grade II, Ib, IV, III) with culture; *A. vaginae* was detected in all women with qPCR, but in none with culture; *G. vaginalis* was detected in all 8 women with qPCR and in 6 women with culture; and *P. bivia* was detected in 5 women with qPCR and in only 2 women with culture.

In summary, qPCR was more sensitive than culture, whereas culture detected only high concentrations of bacteria or failed to detect fastidious bacteria, such as *A. vaginae* and *P. bivia*. For research purposes, the qPCR results are of interest to show that BV associated species are present in low numbers in normal VMF. Culture remains useful for diagnostic purposes to some degree, because it detects the most abundant species, associated with normal or disturbed VMF, but it misses *A. vaginae*, which produces small, slowly growing colonies, which are difficult to detect among the other BV-associated species, and also misses largely *P. bivia*.

### The effect of antibiotic therapy as determined by qPCR and culture

Two women (group D) used antimicrobial agents (not specified) during the course of this study.

Subject #16 followed an antimicrobial cure. Before the cure, *L. iners* was present at log10–log11 cells/ml, culture revealed also the presence of *L. vaginalis* and yeast was detected with Gram stain. During the cure, *L. iners*, *L. vaginalis* and the yeast were eradicated and instead *Finegoldia magna*, *Atopobium parvulum*, *Enterococcus faecalis* and *Staphylococcus epidermidis* were detected with culture and *A. vaginae* on one day with qPCR. These species possibly colonized the vagina from the rectum [55], [56] during the cure. *F. magna*, *A. parvulum*, *E. faecalis* and *S. epidermidis* persisted after treatment, for the rest of the study period as determined by culture. According to qPCR results, *A. vaginae*, *P. bivia* and *L. iners* were detected after the cure and the yeast re-emerged as detected with Gram stain.

Subject #21 followed a 9-day antibiotics cure. Before the antibiotics cure *L. crispatus*, *L. iners*, *A. vaginae*, *G. vaginalis* and *P. bivia* were present as determined by qPCR and *L. gasseri* was also present as determined by culture. During the antibiotics treatment, *L. crispatus* and *L. gasseri* disappeared completely and *Escherichia coli* and *F. magna* appeared, as determined by culture. After the cure, yeast appeared temporarily on Gram stain, and *L. gasseri* and *L. crispatus* only re-emerged post treatment at 2 and 3 weeks, respectively.

These results suggest that antibiotic treatment severely affects the VMF and that (vaginal) probiotics may be recommendable to help restore the VMF after any antimicrobial treatment.

### Comparison of qPCR/culture and pyrosequencing

Recently, Gajer *et al.* [12] published the first longitudinal study by pyrosequencing [12]. Although it is not easy to compare their results to those previously published, because of their use of different categorical names (community state types instead of grades), it is possible to point to several similar findings. The study of Gajer *et al.* [12] and this one could both establish i) strong interindividual variability of the VMF of different women, ii) similar declines of *L. crispatus* during the menses for the women with predominantly *L. crispatus*, iii) mostly in combination with replacement by *L. iners* or by Gram positive cocci, iv) long term stability of the VMF in most women, v) highest stability for women with grade III VMF (when assuming that their community state type (CST) IV-B largely corresponds to grade III VMF or BV-associated VMF).

Gajer *et al.* [12] considered long term stability as another hallmark of normal VMF. However, one should be cautious, because stability does not necessarily equals eubiosis. In our longitudinal study ([10] and this study), we found that women with grade III VMF (*i.e.* BV associated VMF) had even more stable VMF than women with predominantly grade I VMF, and that most of these women were asymptomatic. This should not be considered as normal VMF, because also chronic BV is characterized by a very stable VMF, but it is associated with *G. vaginalis* and *A. vaginae* biofilm formation [57].

In conclusion, the more recent pyrosequencing based microbiome studies [2], [12], [19] have expanded our view on the diversity of the vaginal bacterial population, but is too precocious to state that they come to opposite conclusions with respect to past studies with culture-dependent and culture-independent techniques.

### Conclusions

Despite the temporal, short term, dynamic changes, the VMF of most women in this study displayed long term overall stability. The principle cause of disturbance of the VMF in this study were the menses, in accordance with findings of previous studies. The use of antibiotics was also a major disturbing factor. The number of assessable incidences of sexual intercourse, although showing some influence on the presence of *A. vaginae* and *G. vaginalis*, *P. bivia*, was too low to draw valid conclusions.

Women with grade Ia VMF, *i.e. L. crispatus* dominated VMF, combined long term stability with short term menses-associated fluctuations of the VMF, which entailed the reduction of *L. crispatus* and the strong increase of the BV-associated bacteria (*A. vaginae*, *G. vaginalis* and *P. bivia*) and of *L. iners*, which we previously also found to be strongly associated with BV-associated VMF.

The VMF of women with grade III VMF showed even higher stability than grade I VMF, with constant high concentrations (log8 cells/ml average) of *L. iners*, *A. vaginae*, *G. vaginalis* (predominantly sialidase positive) and *P. bivia*, not changing during menses, and the virtual absence of *L. crispatus*. Only for a limited number of women, frequent short term fluctuations, *i.e.* shifts between grades Ib, II, III and IV VMF, were observed, with high concentrations of both *L. crispatus* and the BV-associated bacteria *L. iners*, *A. vaginae*, *G. vaginalis* and *P. bivia*, possibly indicating continued competition between *L. crispatus* and the BV-associated anaerobes.

The presence of stability in grade I, and possibly even more in grade III VMF was reported in our previous study [10] and this finding was recently reconfirmed in the longitudinal pyrosequencing study by Gajer *et al.* [12].

### Limitations of the study

A qPCR study is limited to the species that are analyzed, which implies that most of the vaginal microflora is not analyzed. However, due to the combination of culture [10] and qPCR, we obtained complementary information, *i.e. A. vaginae* and *P. bivia* that remained undetected in the culture study were detected by qPCR and more women appeared to be colonized by *A. vaginae*, *G. vaginalis, L. iners and P. bivia* with qPCR than could be assessed with culture.

Jellyfish DNA was used as an internal inhibition control, indicating that drops in bacterial concentrations during the menses, as established with five qPCR formats were not due to PCR inhibition, *i.e.* not caused by blood present in the swab and/or DNA-extract. Moreover, according to qPCR, the concentrations of several species increased during or around menses.

No human 18S rRNA gene qPCR was used to measure human DNA levels, verifying that the swab contacted a human tissue surface and that the extraction was successful. However, we mostly analyzed DNA extracts from subsequent days, so these samples could serve as controls for each other and any aberration would not have gone unnoticed.

Not all days of the 2 menstrual cycles were analyzed by qPCR for all women. Although the analyzed days were well chosen, we cannot exclude that interesting observations could have been missed.

Other possible limitations to this paper were reported previously [10].

## Supporting Information

File S1 **Bacterial concentrations of** ***Lactobacillus crispatus*** **,** ***L. iners*** **,** ***Atopobium vaginae*** **,** ***Prevotella bivia*** **and sialidase positive** ***Gardnerella vaginalis*** **during the menstruation cycle.** This is a figure in PDF format. The file can be viewed with Adobe Acrobat reader
(PDF)

File S2 **A detailed overview of all species that colored blue on TMB+ agar.** This is a table in PDF format. The file can be viewed with Adobe Acrobat reader
(PDF)
