# Supplementary material for: Longitudinal qPCR Study of the Dynamics of L. crispatus, L. iners, A. vaginae, (Sialidase Positive) G. vaginalis, and P. bivia in the Vagina
Source: PLoS One. 2012 Sep 21;7(9):e45281. doi: 10.1371/journal.pone.0045281 (PMC3448655; doi:10.1371/journal.pone.0045281)
Supplement: File S1 — Bacterial concentrations of Lactobacillus crispatus , L. iners , Atopobium vaginae , Prevotella bivia and sialidase positive Gardnerella vaginalis during the menstruation cycle. This is a figure in PDF format. The file can be viewed with Adobe Acrobat reader (PDF) [file pone.0045281.s001.pdf]

Supporting Information file 1.

Bacterial concentrations of *Lactobacillus crispatus*, *L. iners*, *Atopobium vaginae*, *Prevotella bivia* and sialidase positive *Gardnerella vaginalis* during the menstruation cycle

Normal VMF Group (N)

Normal VMF

N = 9

Legend

**VMF grade**	1-L: grade I-like			
**H_2O_2 production**	0 : No production	1: weak production	2: strong production	3: very strong production
**GPC (Gram positive cocci)**	G: Gram positive cocci present			
**Yeast**	Y: Yeast present			
**Menses**	M: Menses			
**Sexual intercourse**	C: Condom	NC: No condom		
**Antibiotics**	AB: Antibiotics use			
**Hygiene**	S: shower	B: bath	IH: Intimate hygiene	O: No hygiene reported
**NA**	Not assessed			
**NT**	Not tested due to loss of DNA-extract			

Subject #02

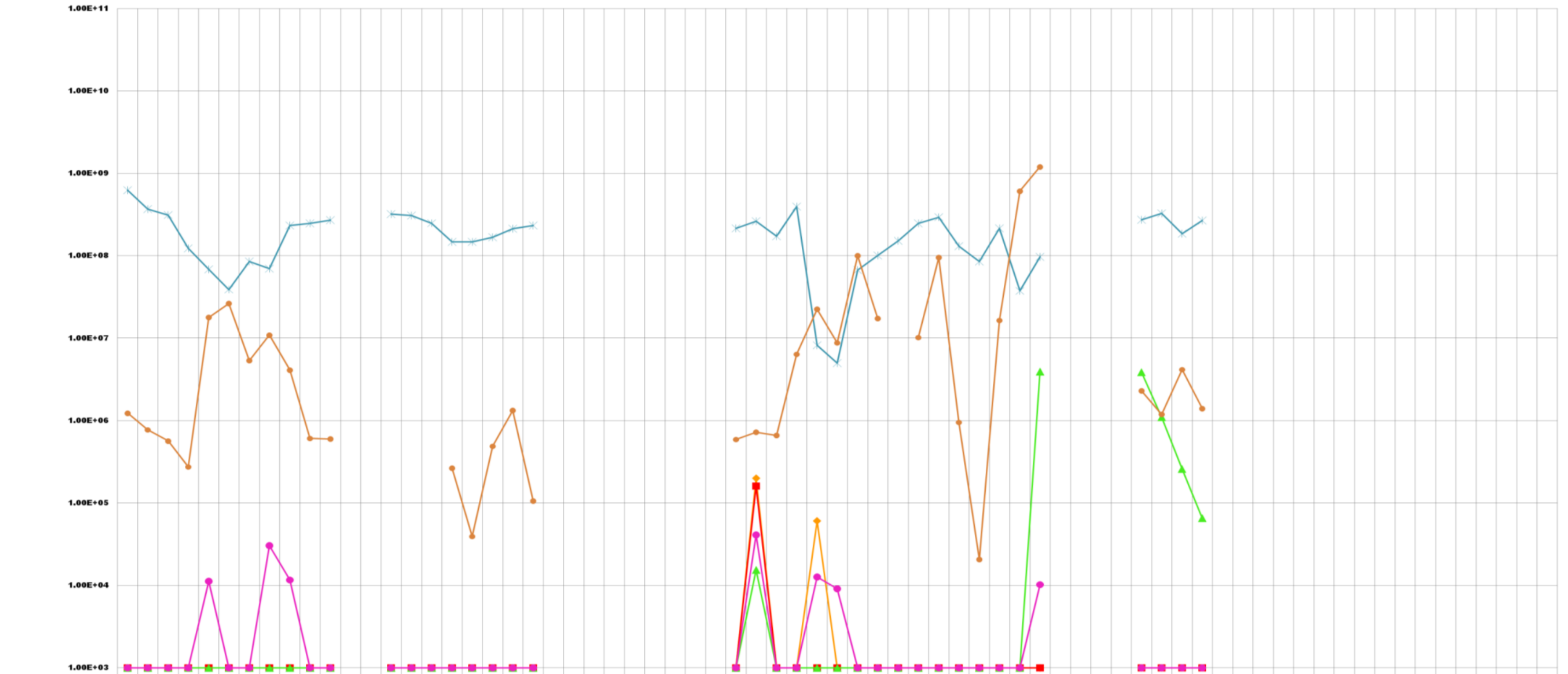

Day	VMF Grade	H_2O_2 score	GPC	Yeast	Menses	Sexual intercourse	Antibiotics	Hygiene
1	Ia			0			0	S
2	Ia							S
3	Ia				M			S
4	Ia				M			S
5	Ia		G		M			S
6	Ia		G		M			S
7	Ia	3	G		M			S
8	Ia		G		M			S
9	Iab				M			S
10	Iab							S
11	Ia							S
12	Ia							S
13	Ia							S
14	Ia	3						S
15	Ia							S
16	Iab							S
17	Ia							S
18	Ia		G					S
19	Ia		G					S
20	Iab							S
21	Iab	3						S
22	Iab							S
23	Iab							S
24	Iab							S
25	Iab							S
26	Iab							0
27	Iab							S
28	Iab	3						S
29	Iab							S
30	Iab							S
31	Iab							S
32	Iab							S
33	Iab				M			S
34	Ia		G		M			S
35	II	1			M			S
36	II				M			S
37	Ia		G		M			S
38	Ia		G					S
39	Iab		G					S
40	Ia							S
41	Iab							S
42	Iab	3				C		S
43	Ia							S
44	Ia							S
45	II							S
46	II							S
47	II							S
48	II							S
49	II	3						S
50	II							S
51	II							S
52	II							S
53	Iab							S
54	Iab							S
55	Iab							S
56	Iab	3						S
57								NA
58								NA
59								NA
60								NA
61								NA
62								NA
63								NA
64								NA
65								NA
66								NA
67								NA
68								NA
69								NA
70								NA
71								NA

Subject #04

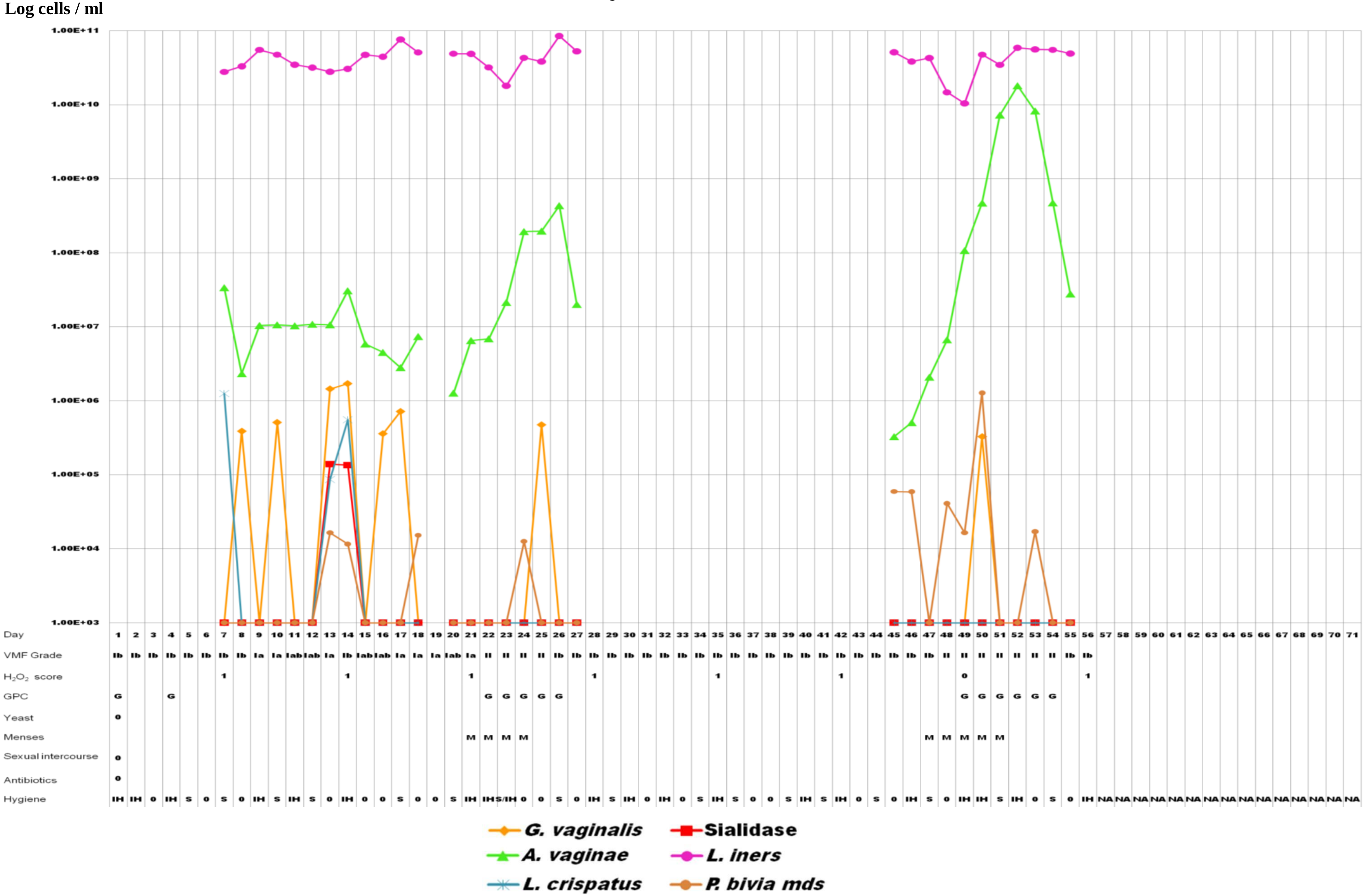
Log cells / ml
1.00E+11
1.00E+10
1.00E+09
1.00E+08
1.00E+07
1.00E+06
1.00E+05
1.00E+04
1.00E+03
Day
VMF Grade
H_2O_2 score
GPC
Yeast
Menses
Sexual intercourse
Antibiotics
Hygiene
G. vaginalis
A. vaginae
L. crispatus
Sialidase
L. iners
P. bivia mds

Subject #05

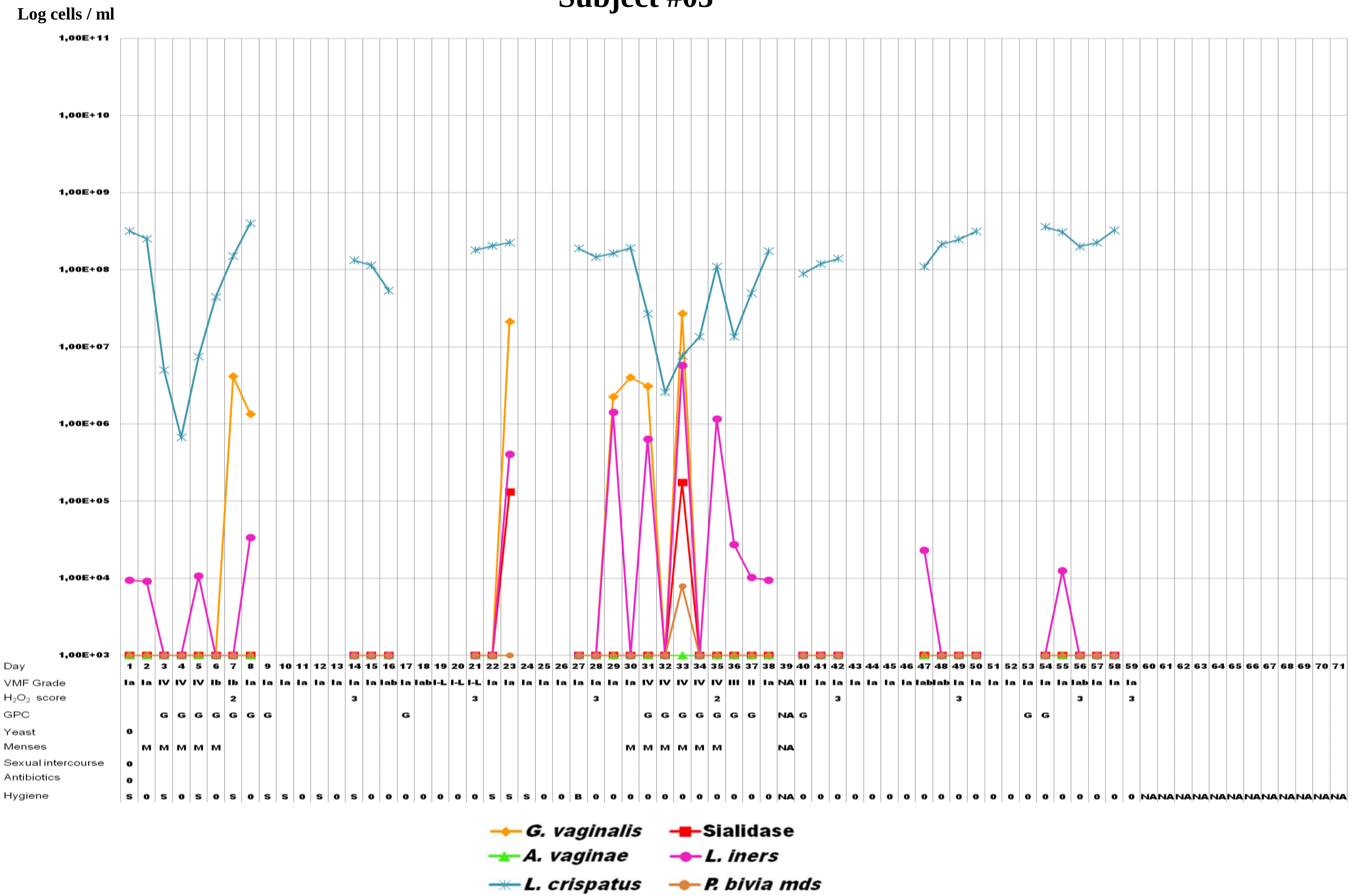

Day	1	2	3	4	5	6	7	8	9	10	11	12	13	14	15	16	17	18	19	20	21	22	23	24
VMF Grade	Ia	Ia	IV	IV	IV	Ib	Ib	Ia	Ia	Ia	Ia	Ia	Ia	Ia	Ia	Iab	Ia	Iab	I-L	I-L	I-L	Ia	Ia	Ia
H_2O_2 score							2							3							3			
GPC			G	G	G	G	G	G	G								G							
Yeast	0																							
Menses		M	M	M	M	M																		
Sexual intercourse	0																							
Antibiotics	0																							
Hygiene	S	0	S	0	S	0	S	0	S	S	0	S	0	S	0	0	0	0	0	0	0	S	S	S

Day	25	26	27	28	29	30	31	32	33	34	35	36	37	38	39	40	41	42	43	44	45	46	47	48
VMF Grade	Ia	Ia	Ia	Ia	Ia	Ia	IV	IV	IV	IV	IV	III	II	Ia	NA	II	Ia	Ia	Ia	Ia	Ia	Ia	Iab	Iab
H_2O_2 score				3							2							3						
GPC							G	G	G	G	G	G	G		NA	G								
Yeast																								
Menses						M	M	M	M	M	M				NA									
Sexual intercourse																								
Antibiotics																								
Hygiene	0	0	B	0	0	0	0	0	0	0	0	0	0	0	NA	0	0	0	0	0	0	0	0	0

Day	49	50	51	52	53	54	55	56	57	58	59	60	61	62	63	64	65	66	67	68	69	70	71
VMF Grade	Ia	Ia	Ia	Ia	Ia	Ia	Ia	Iab	Ia	Ia	Ia												
H_2O_2 score	3							3			3												
GPC					G	G																	
Yeast																							
Menses																							
Sexual intercourse																							
Antibiotics																							
Hygiene	0	0	0	0	0	0	0	0	0	0	0	NA	NA	NA	NA	NA	NA	NA	NA	NA	NA	NA	NA

G. vaginalis **Sialidase**
A. vaginae ***L. iners***
L. crispatus ***P. bivia* mds**

Subject #06

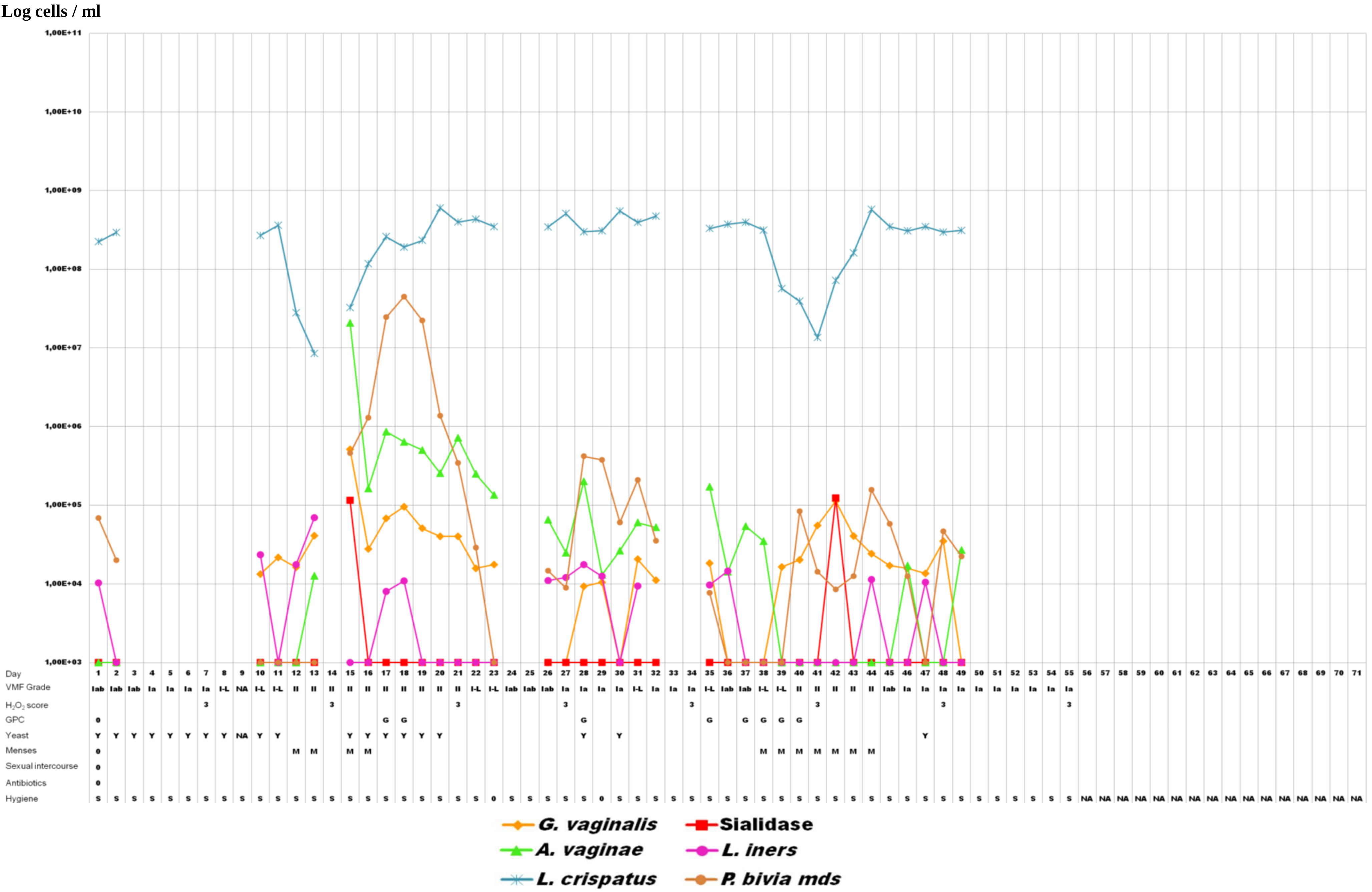

Log cells / ml
1,00E+11
1,00E+10
1,00E+09
1,00E+08
1,00E+07
1,00E+06
1,00E+05
1,00E+04
1,00E+03
Day
VMF Grade
H_2O_2 score
GPC
Yeast
Menses
Sexual intercourse
Antibiotics
Hygiene
G. vaginalis
A. vaginae
L. crispatus
Sialidase
L. iners
P. bivia mds

Subject #12

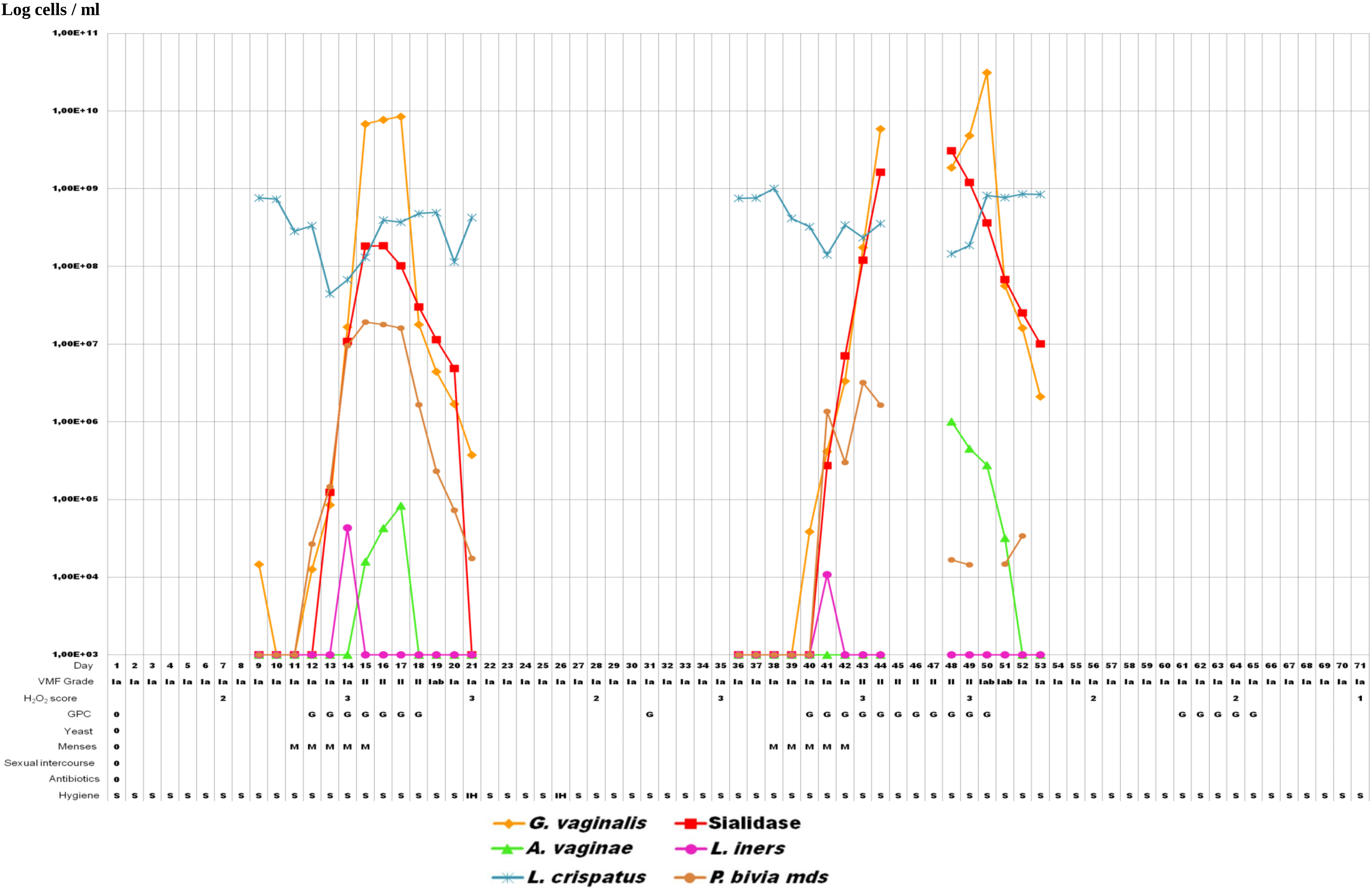

Log cells / ml
1,00E+11
1,00E+10
1,00E+09
1,00E+08
1,00E+07
1,00E+06
1,00E+05
1,00E+04
1,00E+03
Day
VMF Grade
H_2O_2 score
GPC
Yeast
Menses
Sexual intercourse
Antibiotics
Hygiene
G. vaginalis
Sialidase
A. vaginae
L. iners
L. crispatus
P. bivia mds

Subject #14

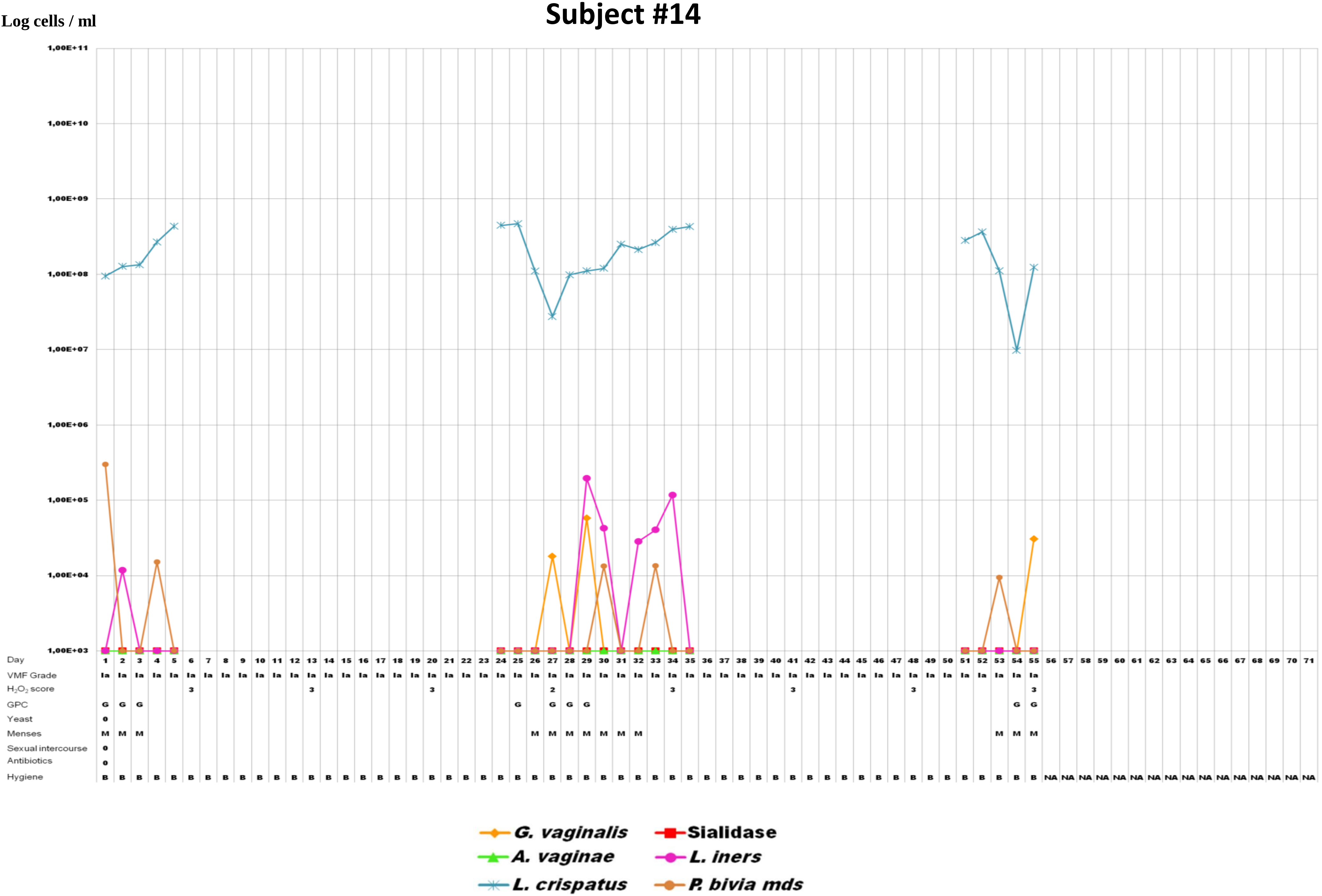

Log cells / ml
1,00E+11
1,00E+10
1,00E+09
1,00E+08
1,00E+07
1,00E+06
1,00E+05
1,00E+04
1,00E+03
Day 1 2 3 4 5 6 7 8 9 10 11 12 13 14 15 16 17 18 19 20 21 22 23 24 25 26 27 28 29 30 31 32 33 34 35 36 37 38 39 40 41 42 43 44 45 46 47 48 49 50 51 52 53 54 55 56 57 58 59 60 61 62 63 64 65 66 67 68 69 70 71
VMF Grade Ia Ia Ia Ia Ia Ia Ia Ia Ia Ia Ia Ia Ia Ia Ia Ia Ia Ia Ia Ia Ia Ia Ia Ia Ia Ia Ia Ia Ia Ia Ia Ia Ia Ia Ia Ia Ia Ia Ia Ia Ia Ia Ia Ia Ia Ia Ia Ia Ia Ia Ia Ia Ia Ia Ia
H_2O_2 score 3 3 3 2 3 3 3 3
GPC G G G G G G G G G
Yeast 0
Menses M M M M M M M M M M M M M
Sexual intercourse 0
Antibiotics 0
Hygiene B B B B B B B B B B B B B B B B B B B B B B B B B B B B B B B B B B B B B B B B B B B B B B B B B B B B B B B NA NA NA NA NA NA NA NA NA NA NA NA NA NA NA NA
G. vaginalis
A. vaginae
L. crispatus
Sialidase
L. iners
P. bivia mds

Subject #15

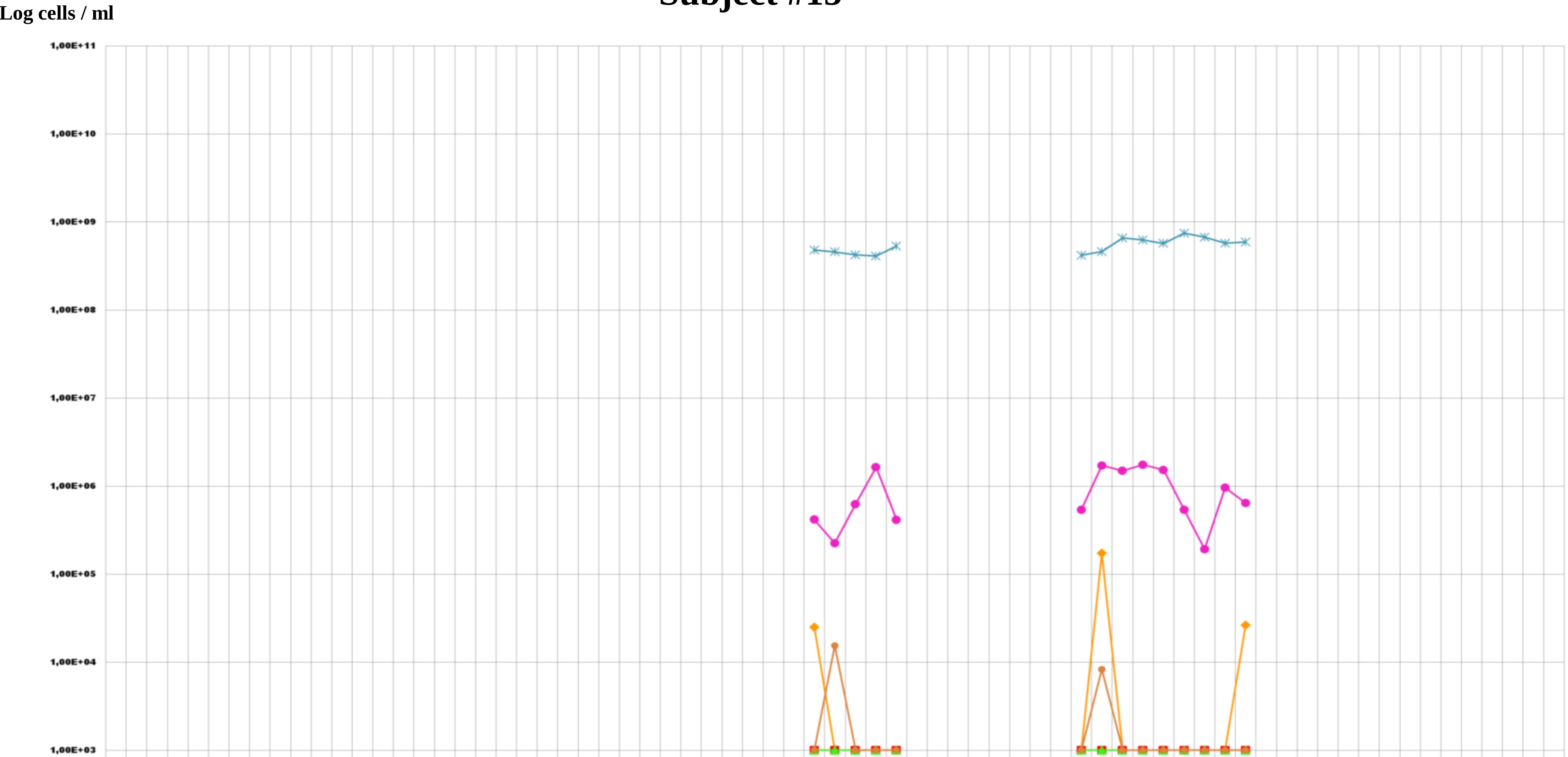

Day	1	2	3	4	5	6	7	8	9	10	11	12	13	14	15	16	17	18
VMF Grade	Ia	Ia	Ia	Ia	Ia	Iab	Ia	Ia	Ia	Ia	Ia	Ia	Ia	Ia	Ia	Ia	Ia	Ia
H_2O_2 score							2							2				
GPC	0																	
Yeast	0																	
Menses	0																	
Sexual intercourse	0																	
Antibiotics	0																	
Hygiene	0	S	0	S	0	0	S	0	S	0	0	S	0	0	S	0	0	S

Day	19	20	21	22	23	24	25	26	27	28	29	30	31	32	33	34	35	36
VMF Grade	Ia	Ia	Ia	Ia	Ia	Ia	Ia	Ia	Ia	Ia	Ia	Ia	Ia	Ia	Ia	Ia	Ia	Ia
H_2O_2 score			2							2							2	
GPC																		
Yeast																		
Menses																		
Sexual intercourse																		
Antibiotics																		
Hygiene	0	S	0	0	S	0	S	S	0	S	0	S	0	S	0	0	S	0

Day	37	38	39	40	41	42	43	44	45	46	47	48	49	50	51	52	53	54
VMF Grade	Iab	Ia	Ia	Ia	Ia	Ia	Ia	Ia	Ia	Ia	Ia	Ia	Ia	Iab	Ia	Ia	Ia	Iab
H_2O_2 score						2							2					
GPC																		
Yeast																		
Menses																		
Sexual intercourse																		
Antibiotics																		
Hygiene	S	0	S	S	0	S	0	S	0	0	S	0	S	0	0	0	S	S

Day	55	56	57	58	59	60	61	62	63	64	65	66	67	68	69	70	71
VMF Grade	Ia	Ia	Ia	Iab	Ia	Ia	Ia	Ia	Ia								
H_2O_2 score		2							2								
GPC																	
Yeast																	
Menses																	
Sexual intercourse																	
Antibiotics																	
Hygiene	0	S	0	S	0	S	0	S	0	NA	NA	NA	NA	NA	NA	NA	NA

Subject #20

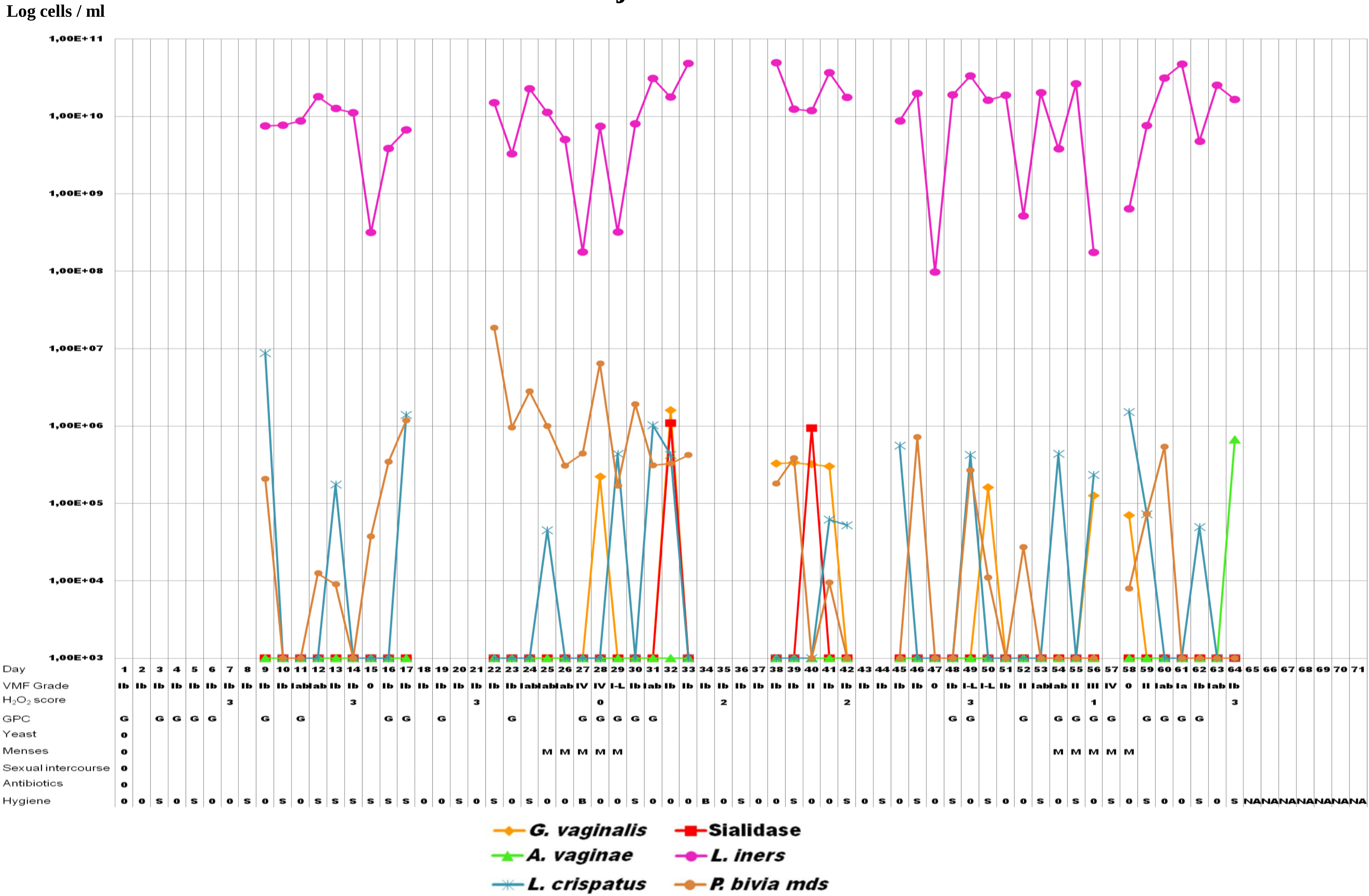

Log cells / ml
1,00E+11
1,00E+10
1,00E+09
1,00E+08
1,00E+07
1,00E+06
1,00E+05
1,00E+04
1,00E+03
Day
VMF Grade
H_2O_2 score
GPC
Yeast
Menses
Sexual intercourse
Antibiotics
Hygiene
G. vaginalis
A. vaginae
L. crispatus
Sialidase
L. iners
P. bivia mds

Subject #22

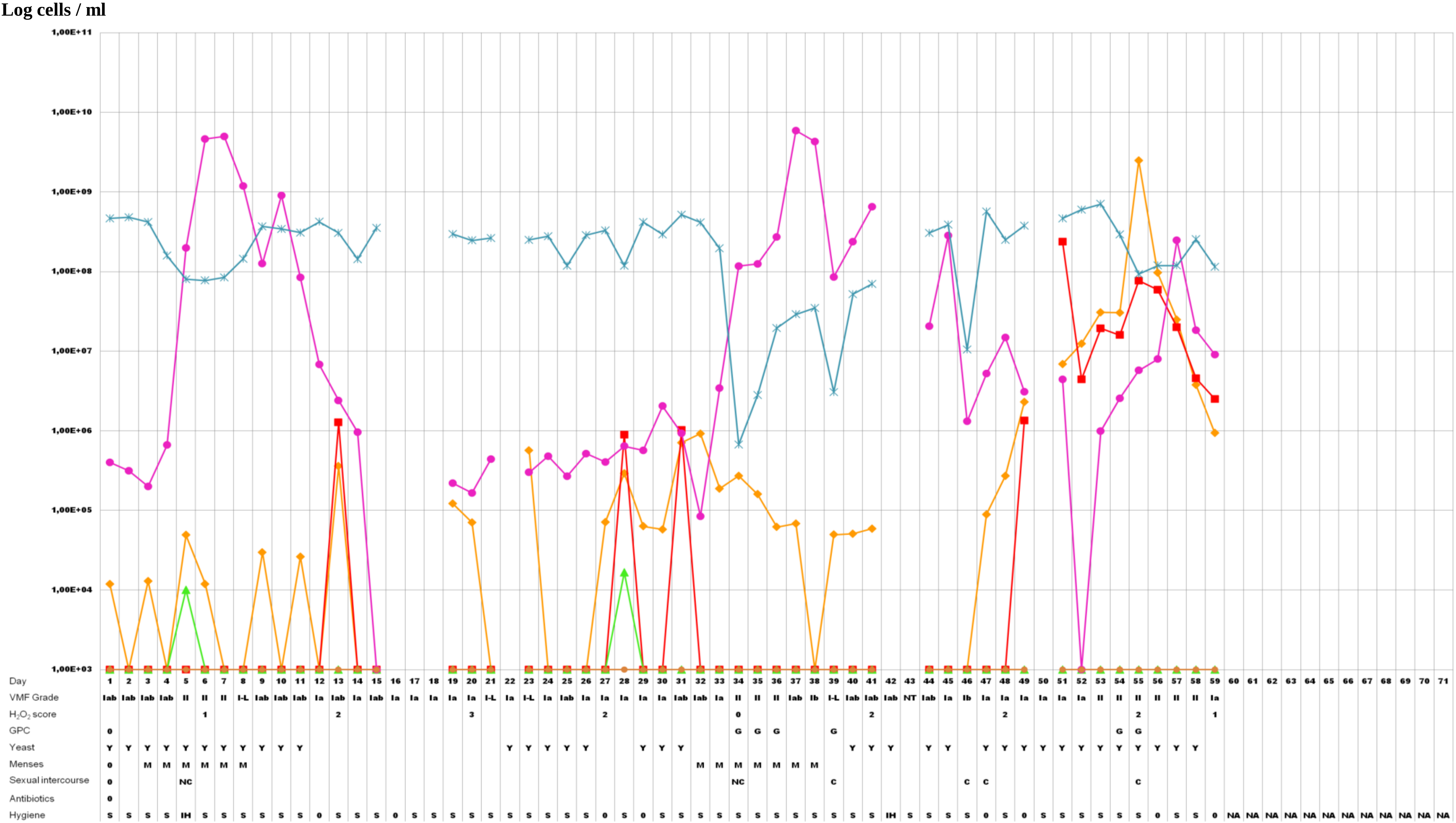

Day	1	2	3	4	5	6	7	8	9	10	11	12	13	14	15	16	17	18	19	20	21	22	23	24
VMF Grade	Iab	Iab	Iab	Iab	II	II	II	I-L	Iab	Iab	Iab	Ia	Iab	Ia	Iab	Ia	Ia	Ia	Ia	Ia	I-L	Ia	I-L	Ia
H_2O_2 score						1							2							3				
GPC	0																							
Yeast	Y	Y	Y	Y	Y	Y	Y	Y	Y	Y	Y											Y	Y	Y
Menses	0		M	M	M	M	M	M																
Sexual intercourse	0				NC																			
Antibiotics	0																							
Hygiene	S	S	S	S	IH	S	S	S	S	S	S	0	S	S	S	0	S	S	S	S	S	S	S	S

Day	25	26	27	28	29	30	31	32	33	34	35	36	37	38	39	40	41	42	43	44	45	46	47	48
VMF Grade	Iab	Ia	Ia	Ia	Ia	Ia	Iab	Iab	Ia	II	II	II	Iab	Ib	I-L	Iab	Iab	Iab	NT	Iab	Ia	Ib	Ia	Ia
H_2O_2 score			2							0							2							2
GPC										G	G	G			G									
Yeast	Y	Y			Y	Y	Y									Y	Y	Y		Y	Y		Y	Y
Menses								M	M	M	M	M	M	M										
Sexual intercourse										NC					C							C	C	
Antibiotics																								
Hygiene	S	S	0	S	0	S	S	S	S	S	S	S	S	S	S	S	S	IH	S	S	S	S	0	S

Day	49	50	51	52	53	54	55	56	57	58	59	60	61	62	63	64	65	66	67	68	69	70	71
VMF Grade	Ia	Ia	Ia	Ia	II	II	II	II	II	II	Ia												
H_2O_2 score							2				1												
GPC						G	G																
Yeast	Y	Y	Y	Y	Y	Y	Y	Y	Y	Y													
Menses																							
Sexual intercourse							C																
Antibiotics																							
Hygiene	0	S	S	S	S	S	S	0	S	S	0	NA	NA	NA	NA	NA	NA	NA	NA	NA	NA	NA	NA

Disturbed VMF Group (D)

Disturbed VMF

N = 8

Subject #13

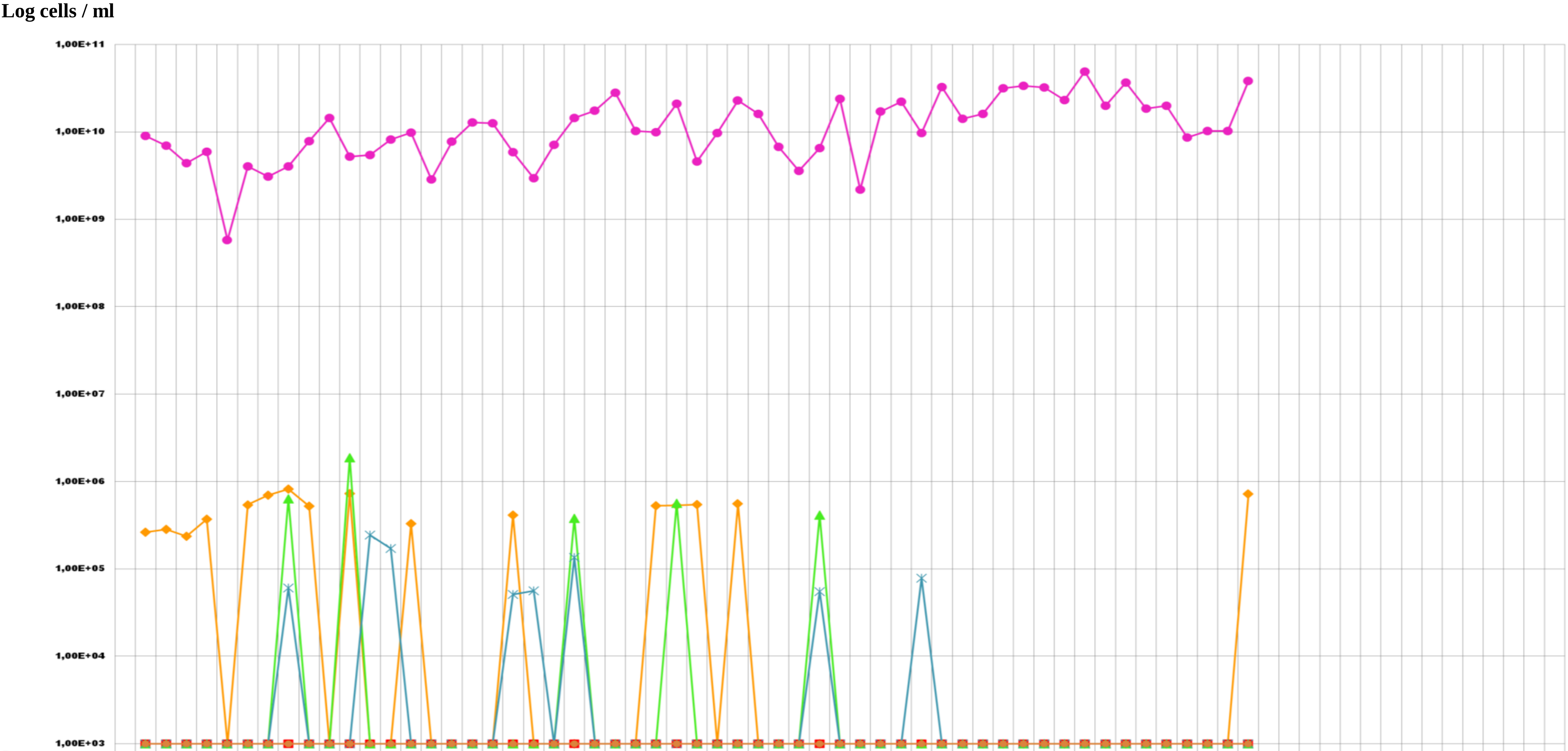

Day	1	2	3	4	5	6	7	8	9	10	11	12	13	14	15	16	17	18	19	20	21	22	23	24
VMF Grade	IV	IV	IV	IV	IV	IV	IV	IV	Ib	Ib	Ib	IV	Ib	IV	IV	IV	IV	Ib	Ib	Ib	IV	IV	Ib	Ib
H_2O_2 score						0							0							1				
GPC	G	G	G	G	G	G	G	G	G	G	G	G	G	G	G	G	G	G	G	G	G	G	G	G
Yeast	0																							
Menses	0			M	M	M	M																	
Sexual intercourse	0										NC								NC			NC		
Antibiotics	0																							
Hygiene	B	0	B	B	B	IH	B	IH	0	0	B	IH	IH	IH	0	B	IH	IH	B	IH	IH	IH	0	B

Day	25	26	27	28	29	30	31	32	33	34	35	36	37	38	39	40	41	42	43	44	45	46	47	48
VMF Grade	Ib	IV	II	Ib	Ib	IV	IV	IV	II	IV	IV	Ib	Ib	Ib	Ib	Ib	Ib	Ib	Ib	Ib	Ib	Ib	II	Ib
H_2O_2 score			1							1							1							0
GPC	G	G	G	G	G	G	G	G	G	G	G	G	G	G	G	G	G	G	G	G	G	G	G	
Yeast																								
Menses								M	M	M	M													
Sexual intercourse			NC				NC						NC		NC			NC				NC		
Antibiotics																								
Hygiene	0	IH	IH	IH	0	B	IH	IH	IH	IH	IH	B	B	0	IH	B	IH	B	IH	IH	0	B	B	IH

Day	49	50	51	52	53	54	55	56	57	58	59	60	61	62	63	64	65	66	67	68	69	70	71
VMF Grade	Ib	Ib	Ib	Ib	Ib	IV	Ib	Ib															
H_2O_2 score								1															
GPC		G	G	G	G	G	G																
Yeast						Y	Y																
Menses																							
Sexual intercourse				NC																			
Antibiotics																							
Hygiene	IH	IH	0	0	B	IH	IH	IH	NA	NA	NA	NA	NA	NA	NA	NA	NA	NA	NA	NA	NA	NA	NA

G. vaginalis — Sialidase
A. vaginae — *L. iners*
L. crispatus — *P. bivia mds*

Subject #16

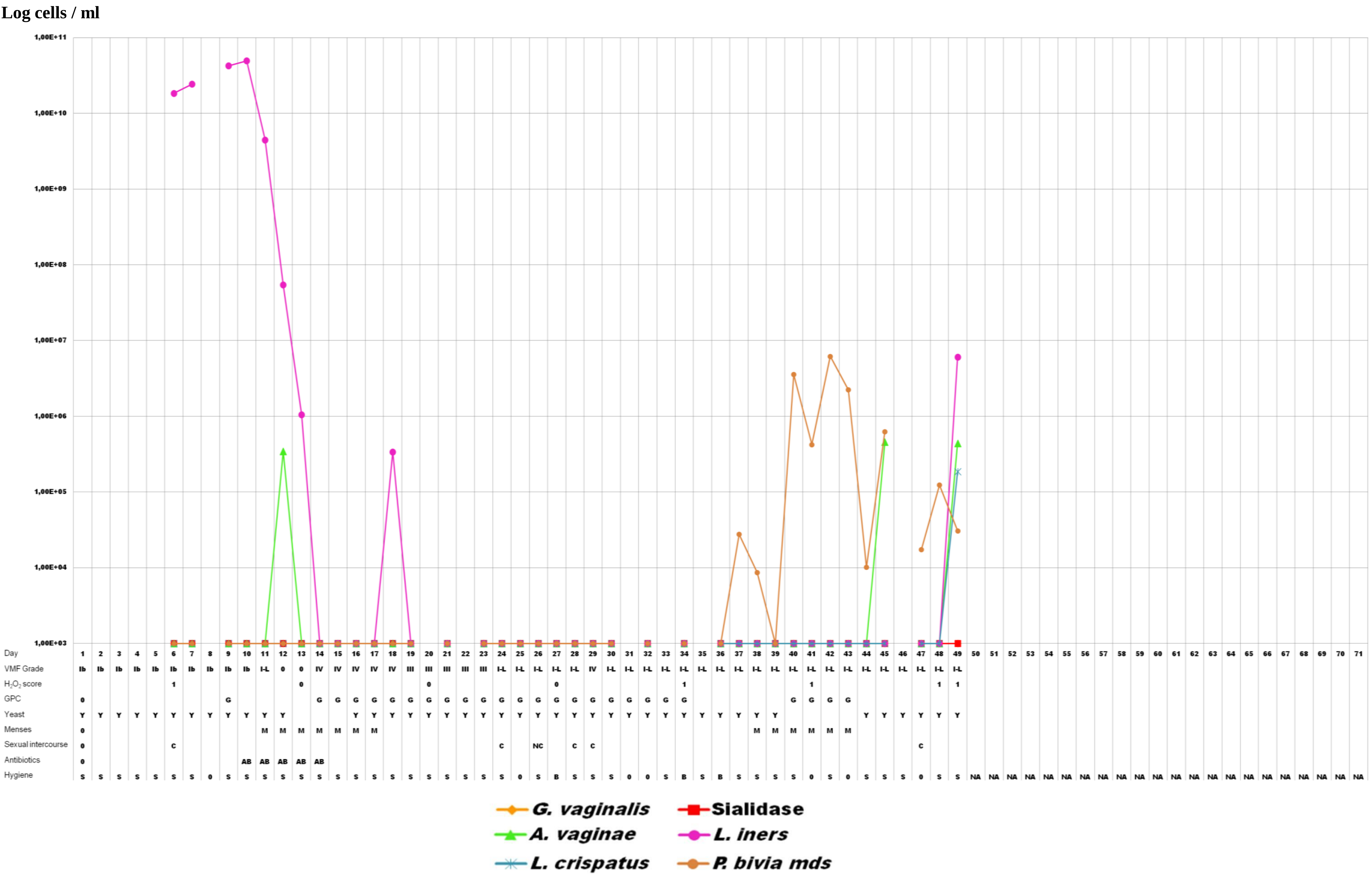
Log cells / ml
1,00E+11
1,00E+10
1,00E+09
1,00E+08
1,00E+07
1,00E+06
1,00E+05
1,00E+04
1,00E+03
Day
VMF Grade
H_2O_2 score
GPC
Yeast
Menses
Sexual intercourse
Antibiotics
Hygiene
G. vaginalis
A. vaginae
L. crispatus
Sialidase
L. iners
P. bivia mds

Subject #18

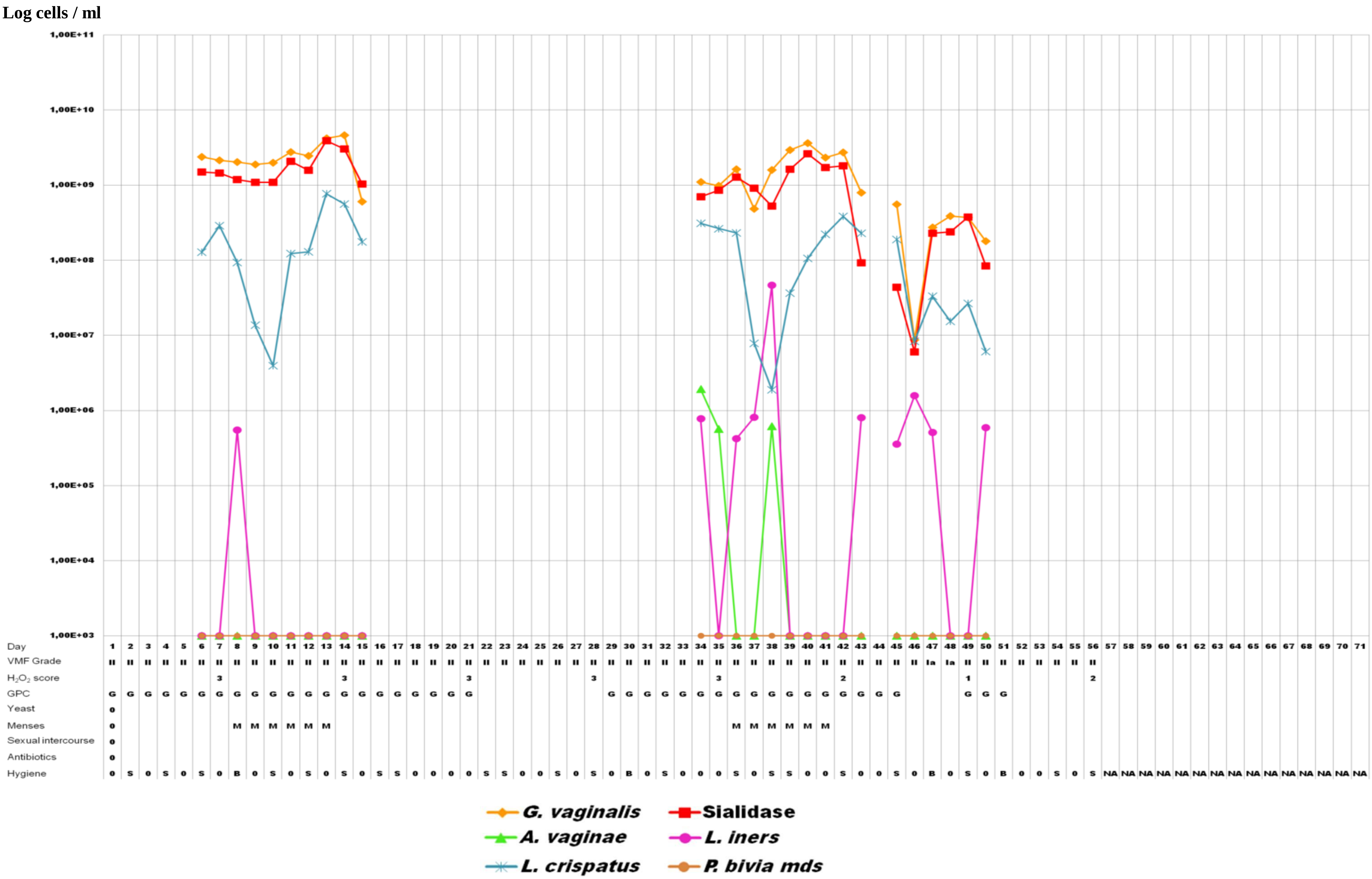
Log cells / ml
1,00E+11
1,00E+10
1,00E+09
1,00E+08
1,00E+07
1,00E+06
1,00E+05
1,00E+04
1,00E+03
Day
VMF Grade
H_2O_2 score
GPC
Yeast
Menses
Sexual intercourse
Antibiotics
Hygiene
G. vaginalis
Sialidase
A. vaginae
L. iners
L. crispatus
P. bivia mds

Subject #19

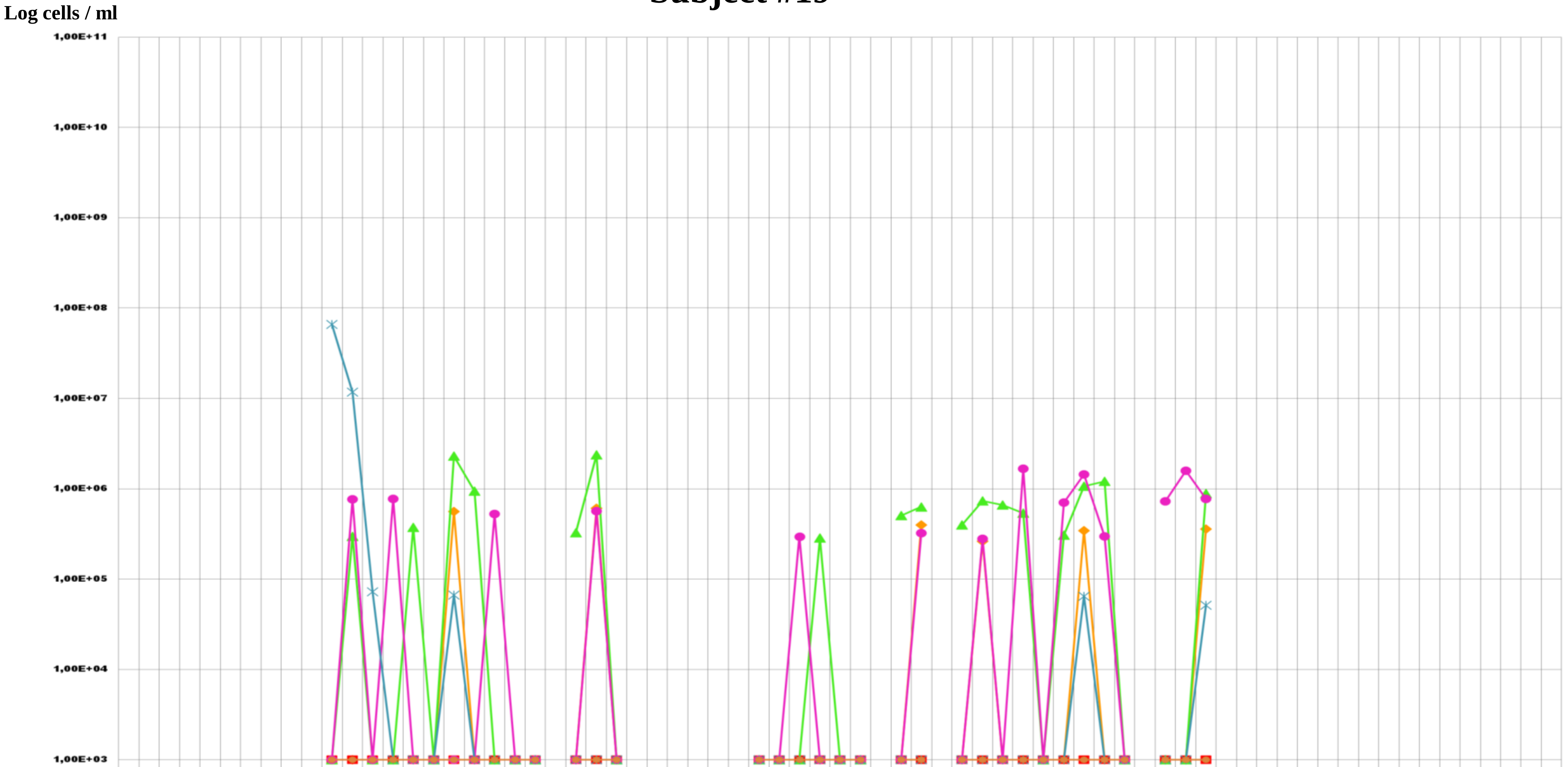

Day	1	2	3	4	5	6	7	8	9	10	11	12	13	14	15	16	17	18	19	20	21	22	23	24
VMF Grade	I-L	I-L	I-L	I-L	I-L	I-L	I-L	I-L	I-L	I-L	I-L	I-L	IV	IV	IV	IV	IV	IV	IV	IV	IV	NT	IV	IV
H_2O_2 score						0							1							1				
GPC	G	G	G	G	G	G	G	G	G	G	G	G	G	G	G	G	G	G	G	G	G	NT	G	G
Yeast	0																							
Menses	0												M	M	M	M	M	M	M			NT		
Sexual intercourse	0											C												
Antibiotics	0																							
Hygiene	S	0	0	S	S	0	S	IH	S	IH	S	IH	IH	S	IH	S	IH	S	IH	IH	IH	NT	IH	IH

Day	25	26	27	28	29	30	31	32	33	34	35	36	37	38	39	40	41	42	43	44	45	46	47	48
VMF Grade	IV	IV	IV	IV	IV	IV	IV	IV	IV	IV	IV	IV	IV	IV	IV	IV	IV	IV	IV	I-L	I-L	I-L	I-L	III
H_2O_2 score					0							0					0							0
GPC	G	G	G	G	G	G	G	G	G	G	G	G	G	G	G	G	G	G	G	G	G	G	G	G
Yeast																								
Menses																				M	M	M	M	
Sexual intercourse																								
Antibiotics																								
Hygiene	S	0	0	S	IH	IH	S	IH	IH	S	0	S	IH	S	IH	0	S	IH	IH	S	IH	0	S	IH

Day	49	50	51	52	53	54	55	56	57	58	59	60	61	62	63	64	65	66	67	68	69	70	71
VMF Grade	IV	Ib	Ib	Ib	IV	IV	IV	IV	IV														
H_2O_2 score									1														
GPC	G	G	G	G	G	G	G	G	G														
Yeast																							
Menses																							
Sexual intercourse					NC																		
Antibiotics																							
Hygiene	IH	IH	S	S	IH	0	S	IH	S	NA	NA	NA	NA	NA	NA	NA	NA	NA	NA	NA	NA	NA	NA

G. vaginalis — Sialidase
A. vaginae — *L. iners*
L. crispatus — *P. bivia mds*

Subject #21

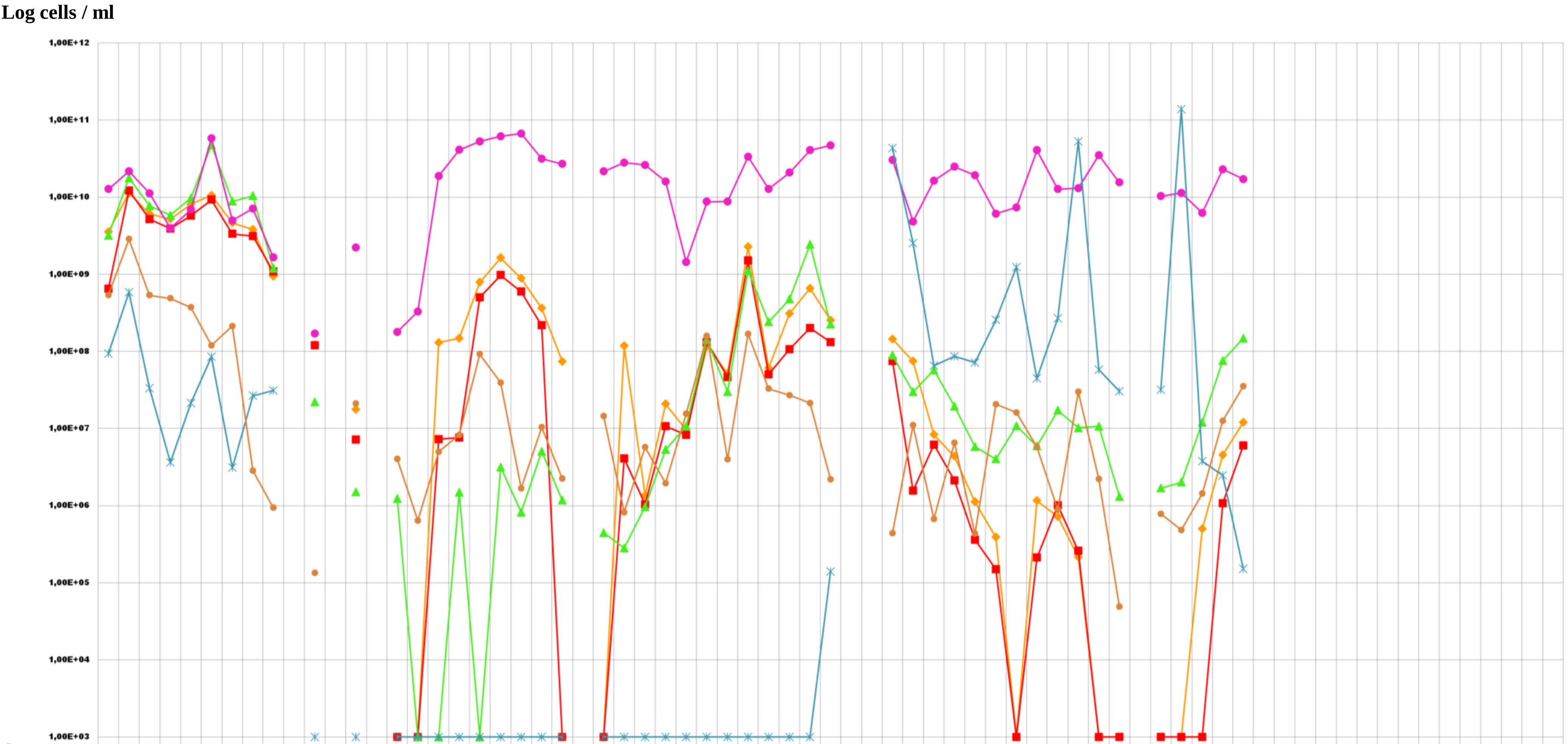

Day	1	2	3	4	5	6	7	8	9	10	11	12	13	14	15	16	17	18	19	20	21	22	23
VMF Grade	II	II	II	III	II	II	III	II	0	0	0	0	0	0	0	0	II	II	III	II	II	II	Ib
H_2O_2 score							3							0							0		
GPC	G	G	G	G	G	G	G	G						G			G	G	G	G		G	G
Yeast	0															Y	Y	Y	Y				
Menses	M	M																					
Sexual intercourse	0																						
Antibiotics	0						AB	AB	AB	AB	AB	AB	AB	AB	AB								
Hygiene	0	0	S	S	0	S	0	S	0	0	0	S	0	S	S	0	S	0	S	S	0	0	0

Day	24	25	26	27	28	29	30	31	32	33	34	35	36	37	38	39	40	41	42	43	44	45	46	47
VMF Grade	Ib	Ib	Ib	Ib	Ib	IV	II	II	II	II	II	II	Ib	Ib	Ib	Ib	Ib	Iab	Ib	Iab	Iab	Ib	Iab	Iab
H_2O_2 score					0							0							2					
GPC		G	G	G	G	G	G	G	G	G	G	G	G	G		G						G	G	
Yeast																								
Menses				M	M	M	M	M	M	M														
Sexual intercourse																								
Antibiotics																								
Hygiene	S	0	0	B	0	0	0	0	S	0	B	0	0	0	S	0	0	S	0	0	S	0	0	0

Day	48	49	50	51	52	53	54	55	56	57	58	59	60	61	62	63	64	65	66	67	68	69	70	71
VMF Grade	Iab	Ib	Ib	Ib	Ib	Ib	II	II	II															
H_2O_2 score		2							1															
GPC	G				G		G	G	G															
Yeast																								
Menses						M	M	M	M															
Sexual intercourse																								
Antibiotics																								
Hygiene	0	S	0	0	0	0	0	S	0	NA	NA	NA	NA	NA	NA	NA	NA	NA	NA	NA	NA	NA	NA	NA

G. vaginalis — Sialidase
A. vaginae — *L. iners*
L. crispatus — *P. bivia mds*

Subject #09

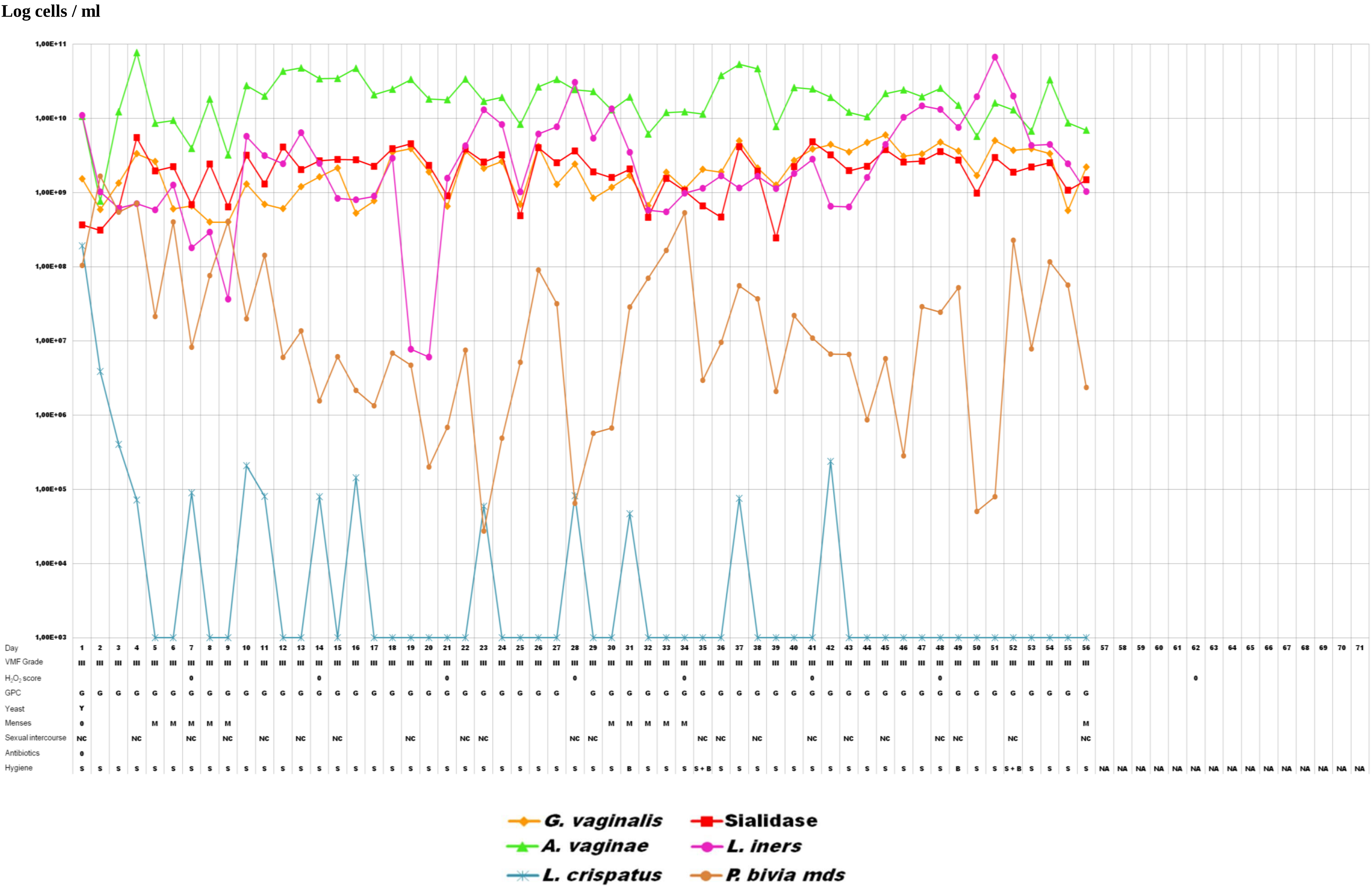
Log cells / ml
1,00E+11
1,00E+10
1,00E+09
1,00E+08
1,00E+07
1,00E+06
1,00E+05
1,00E+04
1,00E+03
Day
VMF Grade
H_2O_2 score
GPC
Yeast
Menses
Sexual intercourse
Antibiotics
Hygiene
G. vaginalis
A. vaginae
L. crispatus
Sialidase
L. iners
P. bivia mds

Subject #24

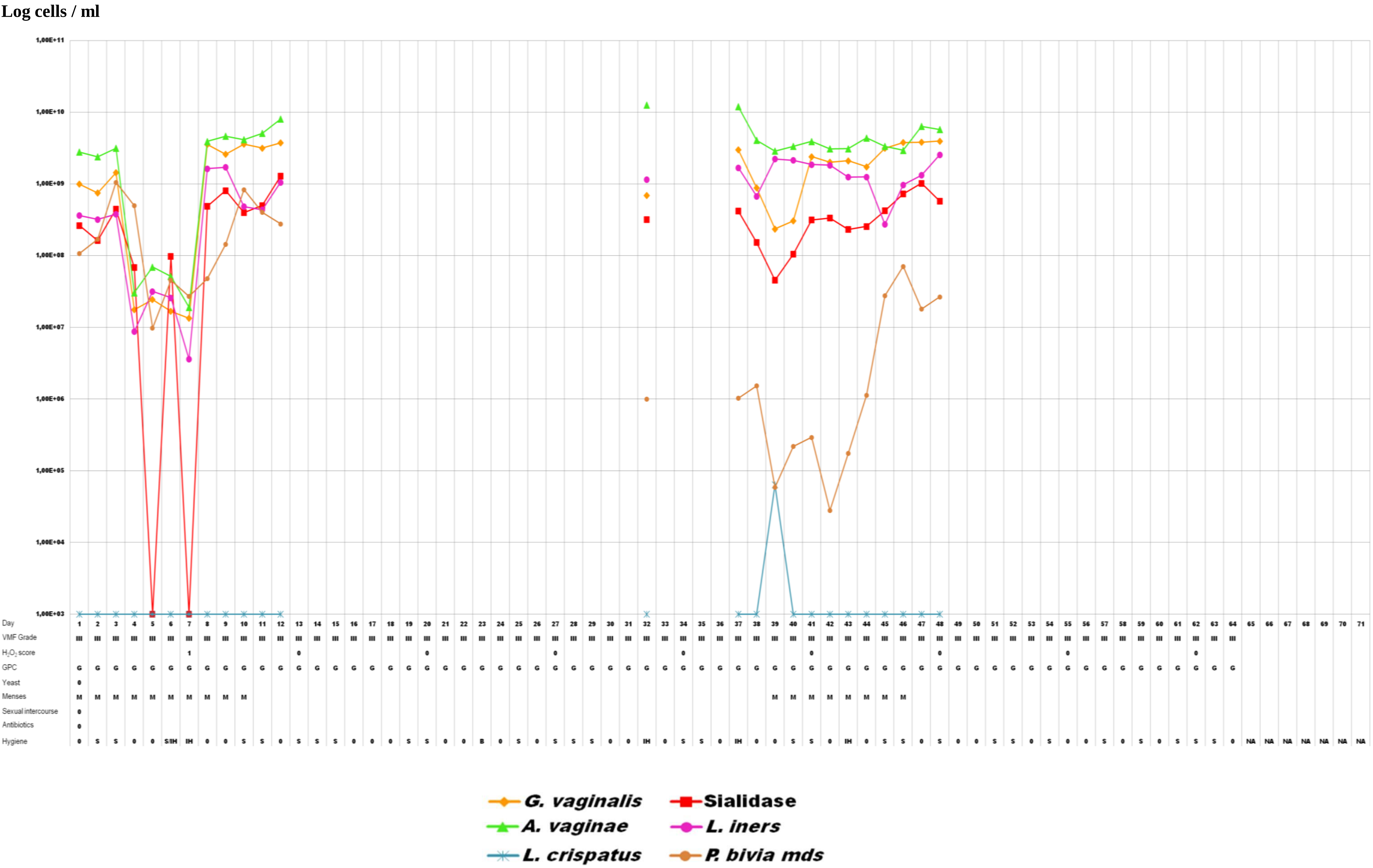
Log cells / ml
1,00E+11
1,00E+10
1,00E+09
1,00E+08
1,00E+07
1,00E+06
1,00E+05
1,00E+04
1,00E+03
Day
VMF Grade
H_2O_2 score
GPC
Yeast
Menses
Sexual intercourse
Antibiotics
Hygiene
G. vaginalis
A. vaginae
L. crispatus
Sialidase
L. iners
P. bivia mds

Subject #25

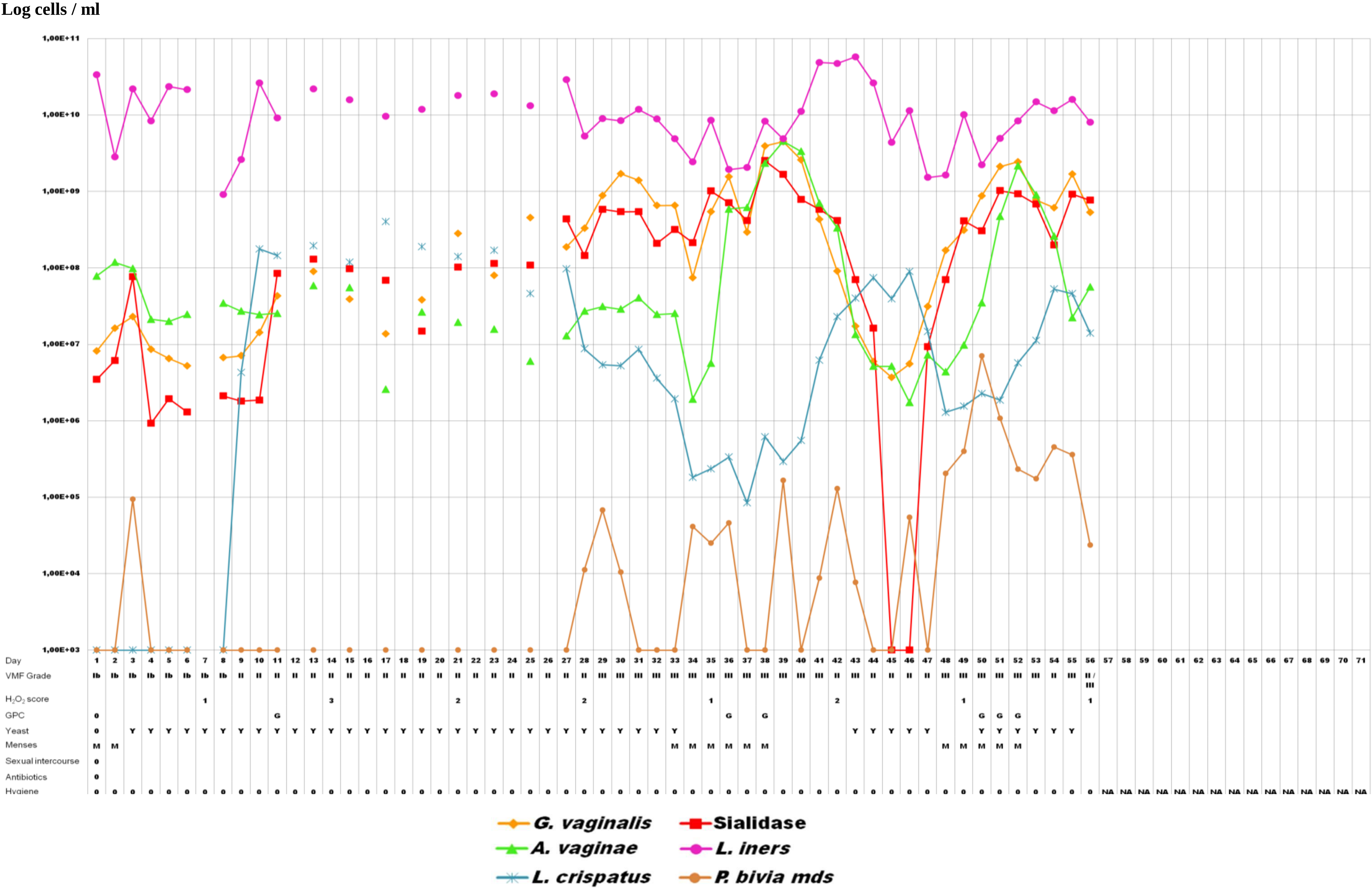

Log cells / ml
1,00E+11
1,00E+10
1,00E+09
1,00E+08
1,00E+07
1,00E+06
1,00E+05
1,00E+04
1,00E+03
Day
VMF Grade
H_2O_2 score
GPC
Yeast
Menses
Sexual intercourse
Antibiotics
Hygiene
G. vaginalis
A. vaginae
L. crispatus
Sialidase
L. iners
P. bivia mds
