# Supplementary material for: Longitudinal qPCR Study of the Dynamics of L. crispatus, L. iners, A. vaginae, (Sialidase Positive) G. vaginalis, and P. bivia in the Vagina
Source: PLoS One. 2012 Sep 21;7(9):e45281. doi: 10.1371/journal.pone.0045281 (PMC3448655; doi:10.1371/journal.pone.0045281)
Supplement: File S2 — A detailed overview of all species that colored blue on TMB+ agar. This is a table in PDF format. The file can be viewed with Adobe Acrobat reader (PDF) [file pone.0045281.s002.pdf]

Supporting Information file 2.

Group	Subject	Culture moment	VMF Grade	Species	H_2O_2-score*
N	#02	Day 7	Ia	*Streptococcus agalactiae*	3
	#02	Day 7	Ia	*Lactobacillus crispatus*	3
	#02	Day 14	Ia	*Lactobacillus jensenii*	3
	#02	Day 14	Ia	*Lactobacillus crispatus*	3
	#02	Day 21	Iab	*Lactobacillus jensenii*	3
	#02	Day 21	Iab	*Streptococcus agalactiae*	3
	#02	Day 21	Iab	*Lactobacillus crispatus*	3
	#02	Day 28	Iab	*Lactobacillus jensenii*	3
	#02	Day 28	Iab	*Lactobacillus crispatus*	3
	#02	Day 28	Iab	*Staphylococcus epidermidis*	3
	#02	Day 35	II	*Peptostreptococcus anaerobius*	1
	#02	Day 35	II	*Peptoniphilus asaccharolyticus*	1
	#02	Day 35	II	*Streptococcus anginosus* group	1
	#02	Day 35	II	*Alloscardovia omnicolens*	1
	#02	Day 42	Iab	*Streptococcus agalactiae*	3
	#02	Day 42	Iab	*Lactobacillus crispatus*	3
	#02	Day 42	Iab	*Lactobacillus jensenii*	3
	#02	Day 49	II	*Lactobacillus jensenii*	3
	#02	Day 49	II	*Lactobacillus crispatus*	3
	#02	Day 56	Iab	*Lactobacillus jensenii*	3
	#02	Day 56	Iab	*Alloscardovia omnicolens*	3
	#04	Day 7	Ib	*Lactobacillus gasseri*	1
	#04	Day 7	Ib	*Alloscardovia omnicolens*	1
	#04	Day 14	Ib	*Alloscardovia omnicolens*	1
	#04	Day 14	Ib	*Lactobacillus gasseri*	1
	#04	Day 21	Ia	*Lactobacillus gasseri*	1
	#04	Day 35	Ib	*Lactobacillus gasseri*	1
	#04	Day 42	Ib	*Lactobacillus gasseri*	1
	#04	Day 49	II	*Actinomyces urogenitalis*	0
	#04	Day 56	Ib	*Lactobacillus gasseri*	1
	#05	Day 7	Ib	*Staphylococcus aureus*	2
	#05	Day 7	Ib	*Lactobacillus crispatus*	2
	#05	Day 14	Ia	*Lactobacillus crispatus*	3
	#05	Day 21	I-like	*Streptococcus agalactiae*	3
	#05	Day 21	I-like	*Lactobacillus crispatus*	3
	#05	Day 28	Ia	*Lactobacillus crispatus*	3
	#05	Day 28	Ia	*Streptococcus anginosus*	3
	#05	Day 35	IV	*Lactobacillus crispatus*	2

Group	Subject	Culture moment	VMF Grade	Species	H_2O_2-score*
	#05	Day 35	IV	*Streptococcus agalactiae*	2
	#05	Day 35	IV	*Peptoniphilus asaccharolyticus*	2
	#05	Day 42	Ia	*Staphylococcus haemolyticus*	3
	#05	Day 42	Ia	*Lactobacillus vaginalis*	3
	#05	Day 42	Ia	*Lactobacillus crispatus*	3
	#05	Day 49	Ia	*Bifidobacterium breve*	3
	#05	Day 49	Ia	*Staphylococcus epidermidis*	3
	#05	Day 49	Ia	*Lactobacillus vaginalis*	3
	#05	Day 56	Iab	*Alloscardovia omnicolens*	3
	#05	Day 56	Iab	*Peptoniphilus asaccharolyticus*	3
	#05	Day 56	Iab	*Lactobacillus crispatus*	3
	#05	Day 59	Ia	*Lactobacillus crispatus*	3
	#05	Day 59	Ia	*Streptococcus anginosus* group	3
	#06	Day 7	Ia	*Lactobacillus crispatus*	3
	#06	Day 7	Ia	*Lactobacillus jensenii*	3
	#06	Day 14	II	*Lactobacillus crispatus*	3
	#06	Day 21	II	*Lactobacillus crispatus*	3
	#06	Day 21	II	*Lactobacillus jensenii*	3
	#06	Day 27	Ia	*Lactobacillus jensenii*	3
	#06	Day 27	Ia	*Lactobacillus crispatus*	3
	#06	Day 34	Ia	*Streptococcus anginosus*	3
	#06	Day 34	Ia	*Lactobacillus crispatus*	3
	#06	Day 41	II	*Lactobacillus jensenii*	3
	#06	Day 41	II	*Lactobacillus crispatus*	3
	#06	Day 48	Ia	*Lactobacillus jensenii*	3
	#06	Day 48	Ia	*Alloscardovia omnicolens*	3
	#06	Day 55	Ia	*Alloscardovia omnicolens*	3
	#12	Day 7	Ia	*Lactobacillus crispatus*	2
	#12	Day 14	Ia	*Lactobacillus jensenii*	3
	#12	Day 14	Ia	*Streptococcus anginosus* group	3
	#12	Day 14	Ia	*Lactobacillus crispatus*	3
	#12	Day 21	Ia	*Lactobacillus jensenii*	3
	#12	Day 21	Ia	*Lactobacillus crispatus*	3
	#12	Day 28	Ia	*Lactobacillus crispatus*	2
	#12	Day 35	Ia	*Lactobacillus crispatus*	3
	#12	Day 43	II	*Lactobacillus jensenii*	3
	#12	Day 49	II	*Lactobacillus jensenii*	3
	#12	Day 49	II	*Lactobacillus crispatus*	3
	#12	Day 56	Ia	*Lactobacillus jensenii*	2
	#12	Day 64	Ia	*Lactobacillus jensenii*	2

Group	Subject	Culture moment	VMF Grade	Species	H_2O_2-score*
	#12	Day 71	Ia	*Lactobacillus jensenii*	1
	#14	Day 6	Ia	*Lactobacillus jensenii*	3
	#14	Day 13	Ia	*Lactobacillus jensenii*	3
	#14	Day 13	Ia	*Lactobacillus crispatus*	3
	#14	Day 20	Ia	*Lactobacillus jensenii*	3
	#14	Day 27	Ia	*Lactobacillus jensenii*	2
	#14	Day 27	Ia	*Streptococcus anginosus* group	2
	#14	Day 34	Ia	*Lactobacillus jensenii*	3
	#14	Day 41	Ia	*Lactobacillus jensenii*	3
	#14	Day 55	Ia	*Lactobacillus jensenii*	3
	#15	Day 7	Ia	*Lactobacillus coleohominis*	2
	#15	Day 7	Ia	*Staphylococcus epidermidis*	2
	#15	Day 21	Ia	*Staphylococcus hominis*	2
	#15	Day 21	Ia	*Veillonella parvula*	2
	#15	Day 28	Ia	*Lactobacillus crispatus*	2
	#15	Day 35	Ia	*Lactobacillus coleohominis*	2
	#15	Day 42	Ia	*Lactobacillus coleohominis*	2
	#15	Day 49	Ia	*Lactobacillus coleohominis*	2
	#15	Day 63	Ia	*Lactobacillus coleohominis*	2
	#20	Day 7	Ib	*Escherichia coli*	3
	#20	Day 7	Ib	*Lactobacillus jensenii*	3
	#20	Day 14	Ib	*Lactobacillus jensenii*	3
	#20	Day 21	Ib	*Lactobacillus jensenii*	3
	#20	Day 21	Ib	*Alloscardovia omnicolens*	3
	#20	Day 28	IV	*Streptococcus anginosus* group	0
	#20	Day 35	Ib	*Lactobacillus jensenii*	2
	#20	Day 42	Ib	*Escherichia coli*	2
	#20	Day 42	Ib	*Lactobacillus jensenii*	2
	#20	Day 49	I-like	*Lactobacillus jensenii*	3
	#20	Day 56	III	*Streptococcus mitis*	1
	#20	Day 56	III	*Lactobacillus jensenii*	1
	#20	Day 56	III	*Streptococcus oralis*	1
	#20	Day 64	Ib	*Lactobacillus jensenii*	3
	#22	Day 6	II	*Lactobacillus jensenii*	1
	#22	Day 6	II	*Lactobacillus gasseri*	1
	#22	Day 13	Iab	*Lactobacillus gasseri*	2
	#22	Day 13	Iab	*Lactobacillus crispatus*	2
	#22	Day 20	Ia	*Lactobacillus gasseri*	3

Group	Subject	Culture moment	VMF Grade	Species	H_2O_2-score*
	#22	Day 27	Ia	*Lactobacillus jensenii*	2
	#22	Day 34	II	*Streptococcus agalactiae*	0
	#22	Day 41	Iab	*Lactobacillus gasseri*	2
	#22	Day 41	Iab	*Lactobacillus jensenii*	2
	#22	Day 48	Ia	*Lactobacillus crispatus*	2
	#22	Day 55	II	*Lactobacillus gasseri*	2
	#22	Day 59	Ia	*Lactobacillus gasseri*	1
D	#13	Day 6	IV	*Alloscardovia omnicolens*	0
	#13	Day 13	Ib	*Alloscardovia omnicolens*	0
	#13	Day 20	Ib	*Lactobacillus gasseri*	1
	#13	Day 20	Ib	*Alloscardovia omnicolens*	1
	#13	Day 27	II	*Lactobacillus gasseri*	1
	#13	Day 27	II	*Streptococcus anginosus* group	1
	#13	Day 34	IV	*Lactobacillus gasseri*	1
	#13	Day 34	IV	*Alloscardovia omnicolens*	1
	#13	Day 34	IV	*Streptococcus anginosus* group	1
	#13	Day 41	Ib	*Lactobacillus gasseri*	1
	#16	Day 27	I-like	*Streptococcus mitis*	0
	#16	Day 34	I-like	*Streptococcus anginosus* group	1
	#16	Day 41	I-like	*Streptococcus anginosus* group	1
	#18	Day 7	II	*Lactobacillus amylovorus*	3
	#18	Day 14	II	*Lactobacillus crispatus*	3
	#18	Day 21	II	*Lactobacillus crispatus*	3
	#18	Day 28	II	*Lactobacillus crispatus*	3
	#18	Day 28	II	*Streptococcus anginosus* group	3
	#18	Day 35	II	*Lactobacillus jensenii*	3
	#18	Day 35	II	*Streptococcus anginosus* group	3
	#18	Day 42	II	*Lactobacillus acidophilus*	2
	#18	Day 42	II	*Lactobacillus jensenii*	2
	#18	Day 42	II	*Lactobacillus gasseri*	2
	#19	Day 13	IV	*Alloscardovia omnicolens*	1
	#19	Day 13	IV	*Streptococcus anginosus* group	1
	#19	Day 20	IV	*Alloscardovia omnicolens*	1
	#19	Day 20	IV	*Streptococcus anginosus* group	1
	#19	Day 29	IV	*Alloscardovia omnicolens*	0
	#19	Day 36	IV	*Enterococcus faecalis*	0
	#19	Day 43	IV	*Streptococcus anginosus*	0
	#19	Day 48	III	*Alloscardovia omnicolens*	0

Group	Subject	Culture moment	VMF Grade	Species	H_2O_2-score*
	#19	Day 57	IV	*Enterococcus faecalis*	1
	#21	Day 7	III	*Lactobacillus crispatus*	3
	#21	Day 14	0	*Escherichia coli*	0
	#21	Day 21	II	*Streptococcus anginosus* group	0
	#21	Day 28	Ib	*Staphylococcus haemolyticus*	0
	#21	Day 42	Ib	*Lactobacillus crispatus*	2
	#21	Day 42	Ib	*Streptococcus anginosus* group	2
	#21	Day 49	Ib	*Lactobacillus crispatus*	2
	#21	Day 49	Ib	*Streptococcus anginosus* group	2
	#21	Day 56	II	*Staphylococcus epidermidis*	1
	#24	Day 7	III	*Streptococcus anginosus* group	0
	#24	Day 27	III	*Streptococcus anginosus* group	0
	#24	Day 34	III	*Streptococcus anginosus* group	0
	#25	Day 7	Ib	*Lactobacillus vaginalis*	1
	#25	Day 7	Ib	*Staphylococcus aureus*	1
	#25	Day 14	II	*Lactobacillus jensenii*	3
	#25	Day 14	II	*Lactobacillus vaginalis*	3
	#25	Day 21	II	*Lactobacillus jensenii*	2
	#25	Day 28	II	*Lactobacillus jensenii*	2
	#25	Day 28	II	*Lactobacillus jensenii*	2
	#25	Day 35	III	*Lactobacillus jensenii*	1
	#25	Day 35	III	*Streptococcus anginosus* group	1
	#25	Day 42	II	*Lactobacillus jensenii*	2
	#25	Day 42	II	*Lactobacillus vaginalis*	2
	#25	Day 49	III	*Streptococcus anginosus* group	1
	#25	Day 49	III	*Lactobacillus jensenii*	1
	#25	Day 56	II/III	*Lactobacillus jensenii*	1
	#25	Day 56	II/III	*Lactobacillus vaginalis*	1

Legend: The days in red represent the culture moments during the menses

*: The H_2O_2-score represents the score of the entire culture plate: score 0 (no H_2O_2 production), score 1 (weak H_2O_2-production), score 2 (strong H_2O_2-production) and score 3 (very blue, very strong H_2O_2-production)
